# Association of clinical biomarker-based biological age and aging trajectory with cardiovascular disease and all-cause mortality in Chinese adults: a population-based cohort study

**DOI:** 10.1186/s12889-025-22114-7

**Published:** 2025-03-04

**Authors:** Qiaoyun Dai, Huayu Sun, Xueying Yang, Shuohua Chen, Xinyuan Zhang, Zhe Yin, Xiujuan Zhao, Shouling Wu, Zongfu Cao, Yuntao Wu, Xu Ma

**Affiliations:** 1 National Human Genetic Resources Center, National Research Institute for Family Planning, Beijing, China; 2 National Human Genetic Resources Sharing Service Platform, Beijing, China; 3 Department of Cardiology, Kailuan General Hospital, Tangshan, China; 4 Graduate School, North China University of Science and Technology, Tangshan, China

**Keywords:** Biological age, Aging trajectory, Cardiovascular disease, Mortality

## Abstract

**Background:**

Evidence on the association of clinical biomarker-based biological age (BA) with cardiovascular disease (CVD) and mortality remains insufficient, particularly concerning aging trajectories' relationship with these two outcomes.

**Methods:**

Seventy-five thousand five hundred thirty-seven Chinese adults from the Kailuan study who participated in the first checkup (2006–2007) were included. BA was predicted by 32 clinical indicators using deep neural networks models. Aging status was divided into decelerated, accelerated, and normal aging based on BA in the first checkup. Six aging trajectories were developed in the initial three checkups. CVD and mortality were followed up till December 31, 2021.

**Results:**

After adjusting for chronological age, sex, education level, occupation, physical activity, smoking status, alcohol consumption, salt consumption habit, history of hypertension, diabetes, and dyslipidemia, as well as the use of antihypertensive, antidiabetic, and lipid-lowering drugs, Cox proportional hazard models showed that relative to normal aging, accelerated aging was a risk factor for CVD (adjusted hazard ratio [aHR], 1.17 [95% CI 1.11–1.23]) and mortality (aHR, 1.17 [1.12–1.22]), while participants with decelerated aging had a lower risk for CVD (aHR, 0.85 [0.80–0.90]) and mortality (aHR, 0.86 [0.82–0.90]). Relative to low-stable trajectory, other aging trajectories associated with higher risk of CVD and death, and high-stable trajectory associated with the highest risk of CVD (aHR, 1.62 [1.45–1.81]) and mortality (aHR, 1.55 [1.41–1.71]). Relative to high-stable trajectory, high-decreasing trajectory was associated with lower risk of CVD (aHR, 0.76 [0.67–0.86]) and death (aHR, 0.78 [0.70–0.87]), and decreasing-increasing trajectory was associated with lower risk of death (aHR, 0.86 [0.75–0.98]).

**Conclusions:**

Accelerated BA aging is associated with a higher risk of CVD and mortality, whereas decelerated aging is associated with a lower risk compared to normal aging. Those persistently at high aging levels are at the highest risk for both CVD and death; conversely, it is the act of lowering and continually maintaining a reduced aging state that effectively mitigates these risks.

**Supplementary Information:**

The online version contains supplementary material available at 10.1186/s12889-025-22114-7.

## Introduction

Rapid population aging has become a global public health priority, with the global older adults increasing to 761 million (9.6%) in 2021 [1]. Amidst this global trend, China is predicted to have 26.1% of its population aged ≥ 65 by 2050 [2], posing significant challenges. Aging is a key risk factor for numerous non-communicable diseases [3]. Notably, cardiovascular diseases (CVD) and mortality are largely driven by population aging [4–6]. Therefore, aging and aging-related diseases pose great challenges to healthcare systems, necessitating a redefined approach to the healthcare of the aging population aimed at preserving or enhancing functional capacity [7].

Aging is progressing at varying rates among individuals [8]. Thus, identifying appropriate indicators to quantify the aging status is of paramount importance. Recently, biological age (BA) has been advanced as an objective measure of individuals’ overall health condition. Multiple modalities of BA have been developed, such as DNA methylation age [9], inflammatory aging clock [10], metabolomic age [11], proteome age [12], and clinical biomarker-based BA [13]. Clinical biomarker-based BA, derived from clinical and biochemical markers, stands out for its exceptional accessibility, standardization, and practicality. It has been rigorously developed across diverse cohorts using multiple methods (principal component analysis, Klemera Doubal method [KDM], deep neural networks [DNN], etc.) [14–16], consistently showing moderate to strong correlation with chronological age (CA).

Aging index and aging status derived from clinical biomarker-based BA have proven effective in predicting aging-related outcomes [13, 17–21], outperforming models based on CA in discriminating mortality risk [13]. Several studies have indicated that elevated BA or aging index serve as risk factors for CVD and mortality [17, 19], with CVD patients exhibiting higher BA than healthy counterparts [13]. Nonetheless, research on BA’s association with CVD incidence and mortality remains limited. Firstly, most studies examined the association between BA and mortality, with a limited investigation into CVD events. The association of BA with CVD subtypes needs to accumulate more evidence. Secondly, the long-term aging trajectory patterns and their association with the incidence of CVD and mortality are poorly characterized. Only one study identified three homogeneous aging trajectories and examined their associations with mortality [22]. However, no studies have estimated the associations of heterogeneous aging trajectory with CVD and mortality. Thirdly, a previous study has confirmed that the BA developed by DNN can predict the risk of death [23], yet its ability to predict CVD remains unexplored. Additionally, while one study demonstrated BA’s predictive power for mortality across all ages [17], most cohorts focused on specific age ranges, typically excluding young and very elderly individuals [13, 18–22]. Thus, more evidence was necessary on the associations of BA and its trajectory with CVD and mortality which is crucial to develop precision anti-aging strategies, inform adaptive health policies, and ultimately enhance life quality and expectancy in aging societies.

This cohort study, based on approximately 15 years of follow-up within the Kailuan study population, aims to evaluate the associations of the aging status and aging trajectories, constructed using clinical biomarker-based BA derived from DNN models, with the incidence of CVD events as well as all-cause mortality.

## Methods

### Study design and participants

The Kailuan study is a prospective cohort study conducted in the Kailuan community in Tangshan, China, which comprises employees and retirees of the Kailuan Group from a large state-owned enterprise. Detailed design and procedures have been described previously [24]. A total of 101 510 participants aged ≥ 18 years were enrolled from 11 hospitals in the Kailuan community during 2006–2007 and underwent health checkups (including questionnaire assessments, clinical examinations, and laboratory tests). Health checkups were performed biennially, and CVD events and mortality data were annually followed up. In this study, baseline data were derived from the first checkup (2006–2007), and aging trajectories were developed from the first, the second (2008–2009), and the third checkup (2010–2011). The endpoint of follow-up on CVD and mortality was December 31, 2021.

**Fig. 1:**
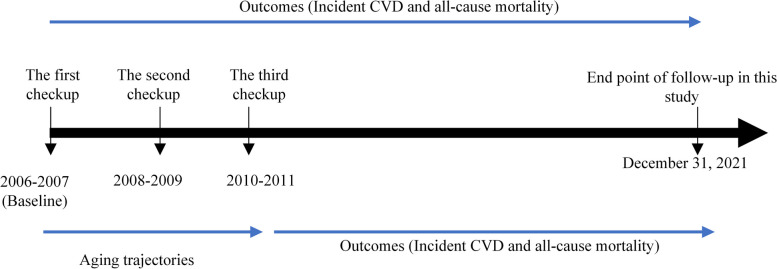
The overview of the study design based on the Kailuan study. CVD indicates cardiovascular disease

**Fig. 2:**
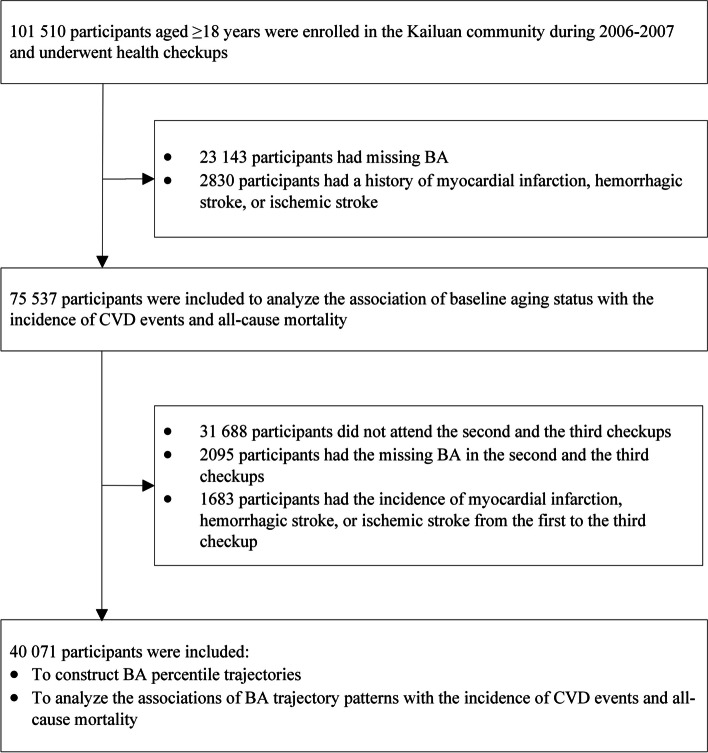
Flowchart for selecting the present study population from the Kailuan study. BA indicates biological age, which was predicted by a biological age model, and the model was constructed using the Deep Neural Networks (DNN) method with 32 clinical and biochemical indicators; CVD indicates cardiovascular disease

Among participants in the first checkup, we excluded those missing BA data or with a history of myocardial infarction, hemorrhagic stroke, or ischemic stroke. This yielded 75 537 participants for analyzing the association of baseline aging status with the incidence of CVD and all-cause mortality. Participants absent from the second and third checkups, missing BA data in these checkups, or experiencing myocardial infarction, hemorrhagic stroke, or ischemic stroke from the first to the third checkup were further excluded. Finally, 40 071 participants formed the cohort for aging trajectories and evaluation of their correlation with CVD and all-cause mortality. Detailed information on the study design and participants is shown in Figs. 1 and 2.

This study conformed to the principles of the Declaration of Helsinki, and written informed consent was obtained from the participants. This study protocol was approved by the Ethics Committee of Kailuan General Hospital (protocol No.: 2021012). The Kailuan study was approved by the Ethics Committee of Kailuan General Hospital, and registered with the Chinese Clinical Trial Registry (Clinical trial number: ChiCTR-TNRC-11001489) on July 19, 2015 (http://www.chictr.org.cn/). This study followed the Strengthening the Reporting of Observational Studies in Epidemiology reporting guideline for cohort studies [25].

### Data collection and variables

The data of the questionnaire assessments, clinical examinations, and laboratory tests were sourced from the Kailuan study database, as previously reported [24, 26, 27]. In this study, 32 clinical and biochemical indicators were used to construct a BA model, and 14 variables from the questionnaire were used as covariates to explore the associations of aging status with CVD and all-cause mortality, as well as those of aging trajectories with CVD and all-cause mortality.

Questionnaire assessments included demographic characteristics, lifestyle, and disease and medication history, as in previous studies [24, 26, 27]. Education (high school or below, college or above), occupation (coal miner, others), smoking status (never, quit, current), alcohol consumption (non-drinker, quit, current drinker), and salt consumption habit (prefer less salty, medium, prefer salty) were modeled as categorical variables. Physical activity was stratified into three groups: low-intensity activity (< 10 min/week), moderate-intensity activity (10–80 min/week), and high-intensity activity (> 80 min/week). Disease history included self-reported conditions such as hypertension, diabetes, and dyslipidemia. Medication usage included regular usage of antihypertensive, antidiabetic, and lipid-lowering drugs.

Clinical indicators included height, weight, waist circumference, hip circumference, and blood pressure were measured by uniformly trained health staff. Venous blood samples were collected in the morning after ≥ 8 h of fasting. Blood biochemical tests were measured on the Hitachi 747 automated analyzer (Hitachi 747; Hitachi, Tokyo, Japan) in the central laboratory of Kailuan General Hospital. Blood routine tests were measured on the automatic hematology analyzer (Sysmex XT-1800i; Sysmex, Kobe, Japan) in the clinical laboratory of each hospital. The instruments used in all hospitals were of the same model, calibrated uniformly, reagents purchased uniformly, and medical staff trained uniformly.

### Biological age and aging status

We constructed BA models using previously reported DNN models [28, 29]. BA prediction was treated as a regression task in which the model takes a vector of indicators and returns a single value of the participant's CA. Indicators were selected from the Kailuan study database that were known to indicate the function, structure, and/or general health of the cardiovascular, hepatic, renal, immune, and metabolic systems, which resulted in 32 clinical and biochemical indicators. Indicators are as follows: (1) Cardiovascular indicators: systolic blood pressure and diastolic blood pressure; (2) Metabolic indicators: body mass index, waist to hip ratio, fasting blood glucose, total cholesterol, triglyceride, high density lipoprotein cholesterol, low density lipoprotein cholesterol; (3) Hepatic indicators: alanine aminotransferase, total bilirubin in blood; (4) Renal indicators: creatinine, blood urea nitrogen, uric acid; (5) Immune indicators: hypersensitive C-reactive protein and blood hematology tests of leukocytes, erythrocytes, thrombocytes, and hemoglobin. Notably, total protein, albumin, and direct bilirubin were not included because they were only available in the third checkup. Detailed information on data preprocessing and indicator selection is shown in the Supplemental methods*.*

DNN model with multiple layers allows fitting data with a high degree of dependencies between the input features (32 indicators) and the output feature (BA). Detailed information on model construction is shown in Supplemental Methods. Briefly, we used the data of the first, second, and third checkups to construct BA models. Then, we used the models from each checkup to predict participants’ BA for subsequent analysis.

Participants were ranked by BA within each CA stratification, and their corresponding percentiles (defined as BA percentile) were calculated. Considering the small sample sizes for CA < 26 and CA ≥ 76, these two portions of participants were classified into two stratifications, respectively; the remaining participants were stratified by CA ∈ [i, i + 1). At each CA stratification in the first checkup, baseline aging status was categorized into decelerated (BA < Q1), normal (Q1 ≤ BA ≥ Q3), or accelerated (BA > Q3) aging groups using BA interquartile range.

Notably, the BA model construction, percentile calculation, and aging status definition mentioned above were sex-specific.

### Aging trajectory assessment

BA percentile in the first, second and third checkup were predicted, respectively. Aging trajectories were identified by the group-based trajectory modeling approach using BA percentile [30], within the SAS PROC TRAJ procedure, as detailed previously [26]. Firstly, models with 2–6 trajectory groups were fitted, with six groups selected as optimal based on the lowest absolute Bayesian Information Criterion (BIC). Then, linear and quadratic functions were tested to identify best-fitting trajectory models. Selection criteria included lower absolute BIC, posterior probabilities of group allocation > 0.7, and minimum sample size in each group > 5.0% of the overall population. Finally, the best model comprised six groups with linear, quadratic, linear, quadratic, quadratic, and quadratic functions.

### Assessment of outcomes

The primary outcomes were the incident CVD and all-cause mortality. Like previous studies [26, 27], we used the International Statistical Classification of Diseases and Related Health Problems 10th Revision to identify CVD events, including myocardial infarction (I21), hemorrhagic stroke (I60 and I61), and ischemic stroke (I63). Participants' IDs were linked to the Hospital Discharge Register and the Municipal Social Insurance Institution to collect the incidence data for CVD, which covers all participants in the Kailuan study. To further identify potential CVD events, we reviewed discharge lists from 11 hospitals during 2006–2021 and asked about CVD history through questionnaires at biannual health checkups. Three experienced physician reviewers blinded to the study design reviewed the medical records for all suspected CVD events. Myocardial infarction diagnosis was based on clinical symptoms, serum concentrations of cardiac enzymes and biomarkers, and electrocardiographic results [31]; stroke diagnosis was based on neurological signs, clinical symptoms, neuroimaging tests, and computed tomography or magnetic resonance imaging [32]. Stroke was divided into ischemic stroke and hemorrhagic stroke.

Mortality information was collected by death certificates from Provincial Vital Statistics Office. All-cause death was defined as death caused by any disease (excluding traffic accidents, suicides, homicides, and other unnatural deaths).

### Statistical analysis

Person-years of CVD were computed from either the first checkup date (baseline aging status analysis) or the third checkup date (aging trajectory analysis) to the earliest of: CVD occurrence, death, or follow-up endpoint (Dec. 31, 2021). Kaplan–Meier method was used to estimate CVD probabilities, and log-rank tests were employed to compare inter-group differences. Cox proportional hazard regression models were used to estimate hazard ratios (HRs) and 95% confidence intervals (CIs) for CVD risk associated with baseline aging status or aging trajectory. The proportional hazards assumption was satisfied by checking the scaled Schoenfeld residual plots (Figs. S1 and S2). A three-stage Cox modeling process was adopted. Model 1 adjusted for CA and sex. Model 2 additionally adjusted for education level, occupation, physical activity, smoking status, alcohol consumption, and salt consumption habit. Model 3 further adjusted for history of hypertension, history of diabetes, history of dyslipidemia, antihypertensive drug, antidiabetic drug, and lipid-lowering drug. Additionally, Cox models were employed to examine the associations between baseline aging status or aging trajectory and CVD subtypes: myocardial infarction, hemorrhagic stroke, and ischemic stroke.

Person-years of death were calculated from the first checkup date (baseline aging status analysis) or the third checkup date (aging trajectory analysis) to the earliest of death or follow-up endpoint (Dec. 31, 2021). The methods and models used to estimate the associations between baseline aging status or aging trajectory and mortality were similar to those used for CVD events.

Notably, to maximize statistical power and minimize potential bias that might occur if participants with any missing data were excluded from analyses, we employed multiple imputation using chained equations to impute missing values for covariates. All analyses used imputed datasets, except when the observed complete data were emphasized.

To assess the robustness of our findings, four sensitivity analyses were conducted: Excluding participants with missing values for observed covariates; Excluding participants with CVD or death within the first two years of follow-up to mitigate reverse causality; To minimize potential biases, participants with a self-reported disease history and medication usage that could influence the outcomes were excluded; Employing Fine-Gray competing risk models treating non-CVD deaths as competing risk events for CVD events, to reduce bias due to competing risk. Moreover, aging trajectories were re-established using participants with complete BA data at all three checkups, followed by repeating primary analyses to verify trajectory stability.

A linear mixed-effects model was employed to further explore the trends of 32 indicators over the initial three checkups within the two interested trajectory groups (low-increasing group and high-decreasing group). The Wald Z-tests of the covariance parameters in the model were utilized to determine whether a random intercept model or a random coefficient model was more appropriate for each indicator. To examine the differences in trends for each indicator between the two trajectory groups, an interaction term between checkup time and group was included in the mixed-effects model.

All analyses were performed using SAS, version 9.4 (SAS Institute Inc., Cary, NC). All statistical tests were two-sided, and values of *P* < 0.05 were considered statistically significant.

## Results

### Baseline characteristics and aging trajectories

Characteristics of 75 537 participants at the first checkup are shown in Table 1. The *mean* (*SD*) CA was 51.07 (12.28) years; 59 894 (79.29%) of the participants were males. Participants with decelerated aging, normal aging, and accelerated aging were 18 853 (24.96%), 37 814 (50.06%), and 18 870 (24.98%), respectively. BA, education level, smoking status, salt consumption habit, disease history, and medication usage demonstrated statistically significant (*P* < 0.05) differences among baseline aging statuses, and no significant differences were observed for CA, sex, occupation, physical activity and alcohol consumption.

**Table 1:** Baseline characteristics of the study population in the first checkup according to the baseline aging status

Characteristic	Total participants (*n* = 75 537)	Decelerated aging (*n* = 18 853)	Normal aging (*n* = 37 814)	Accelerated aging (*n* = 18 870)	*P* Value
CA, mean (SD), years^a^	51.07 (12.28)	51.04 (12.26)	51.06 (12.27)	51.10 (12.34)	0.8655
BA, mean (SD), years^a^	51.14 (8.28)	43.67 (5.89)	51.10 (6.34)	58.67 (6.87)	< 0.0001
Sex					
Female	15 643 (20.71)	3894 (20.65)	7844 (20.74)	3905 (20.69)	0.9684
Male	59 894 (79.29)	14 959 (79.35)	29 970 (79.26)	14 965 (79.31)	
Education level, *n (%)*					
High school or below	69 227 (93.39)	17 067 (91.23)	34 810 (93.80)	17 350 (94.75)	< 0.0001
College or above	4901 (6.61)	1640 (8.77)	2300 (6.20)	961 (5.25)	
Missing	1409	146	704	559	
Occupation, *n (%)*					
Coal miner	22 989 (31.05)	5810 (31.10)	11 603 (31.30)	5576 (30.50)	0.1630
Others	51 052 (68.95)	12 874 (68.90)	25 473 (68.70)	12 705(69.50)	
Missing	1496	169	738	589	
Physical activity, *n (%)*					
Low-intensity	6274 (8.48)	1633 (8.73)	3135 (8.47)	1506 (8.25)	0.0568
Moderate-intensity	56 530 (76.41)	14 305 (76.51)	28 337 (76.55)	13 888 (76.05)	
High-intensity	11 176 (15.11)	2760 (14.76)	5548 (14.99)	2868 (15.70)	
Missing	1557	155	794	608	
Smoking status, *n (%)*					
Never	45 245 (61.09)	11 288 (60.34)	22 757(61.37)	11 200 (61.27)	0.0180
Quit	3751 (5.06)	1012 (5.41)	1796 (4.84)	943 (5.16)	
Current	25 071 (33.85)	6406 (34.25)	12 528 (33.79)	6137 (33.57)	
Missing	1470	147	733	590	
Alcohol consumption, *n (%)*					
Non-drinker	44 496 (60.05)	11 237 (60.06)	22 331 (60.20)	10 928(59.74)	0.4531
Quit	2451 (3.31)	650 (3.47)	1211 (3.26)	590 (3.23)	
Current drinker	27 150 (36.64)	6822 (36.46)	13 552 (36.53)	6776 (37.04)	
Missing	1440	144	720	576	
Salt consumption habit, *n (%)*					
Prefer less salty	6550 (8.85)	1793 (9.59)	3184 (8.59)	1573 (8.61)	0.0014
Medium	59 815(80.80)	14 968 (80.05)	30 055 (81.12)	14 792 (80.94)	
Prefer salty	7661 (10.35)	1938 (10.36)	3812 (10.29)	1911 (10.46)	
Missing	1511	154	763	594	
Self-reported hypertension history, *n (%)*					
No	65 872 (88.87)	17 475 (93.44)	32 902 (88.67)	15 495 (84.58)	< 0.0001
Yes	8253 (11.13)	1226 (6.56)	4203 (11.33)	2824 (15.42)	
Missing	1412	152	709	551	
Self-reported diabetes history, n (%)					
No	72 087 (97.17)	18 479 (98.69)	36 000 (96.96)	17 608 (96.06)	< 0.0001
Yes	2097 (2.83)	245 (1.31)	1129 (3.04)	723 (3.94)	
Missing	1353	129	685	539	
Self-reported dyslipidemia history, n (%)					
No	70 410 (94.91)	17 891 (95.54)	35 202 (94.81)	17 317 (94.48)	< 0.0001
Yes	3774 (5.09)	835 (4.46)	1928 (5.19)	1011 (5.52)	
Missing	1353	127	684	542	
Antihypertensive drug, n (%)					
No	65 872 (90.32)	17 475 (94.38)	32 902 (90.23)	15 495 (86.29)	< 0.0001
Yes	7063 (9.68)	1041 (5.62)	3561 (9.77)	2461 (13.71)	
Missing	2602	337	1351	914	
Antidiabetic drug, n (%)					
No	72 233 (97.86)	18 498 (99.08)	36 077 (97.68)	17 658 (96.96)	< 0.0001
Yes	1579 (2.14)	171 (0.92)	855 (2.32)	553 (3.04)	
Missing	1725	184	882	659	
Lipid-lowering drug, n (%)					
No	70 411 (99.32)	17 891 (99.46)	35 203 (99.30)	17 317 (99.19)	0.0084
Yes	485 (0.68)	97 (0.54)	247 (0.70)	141 (0.81)	
Missing	4641	865	2364	1412	

*CA* Indicates chronological age, *BA* Biological age, *SD* Standard deviation

^a^The analysis of variance was used to examine the differences of baseline characteristics among three aging groups; others used chi-square test

**Fig. 3:**
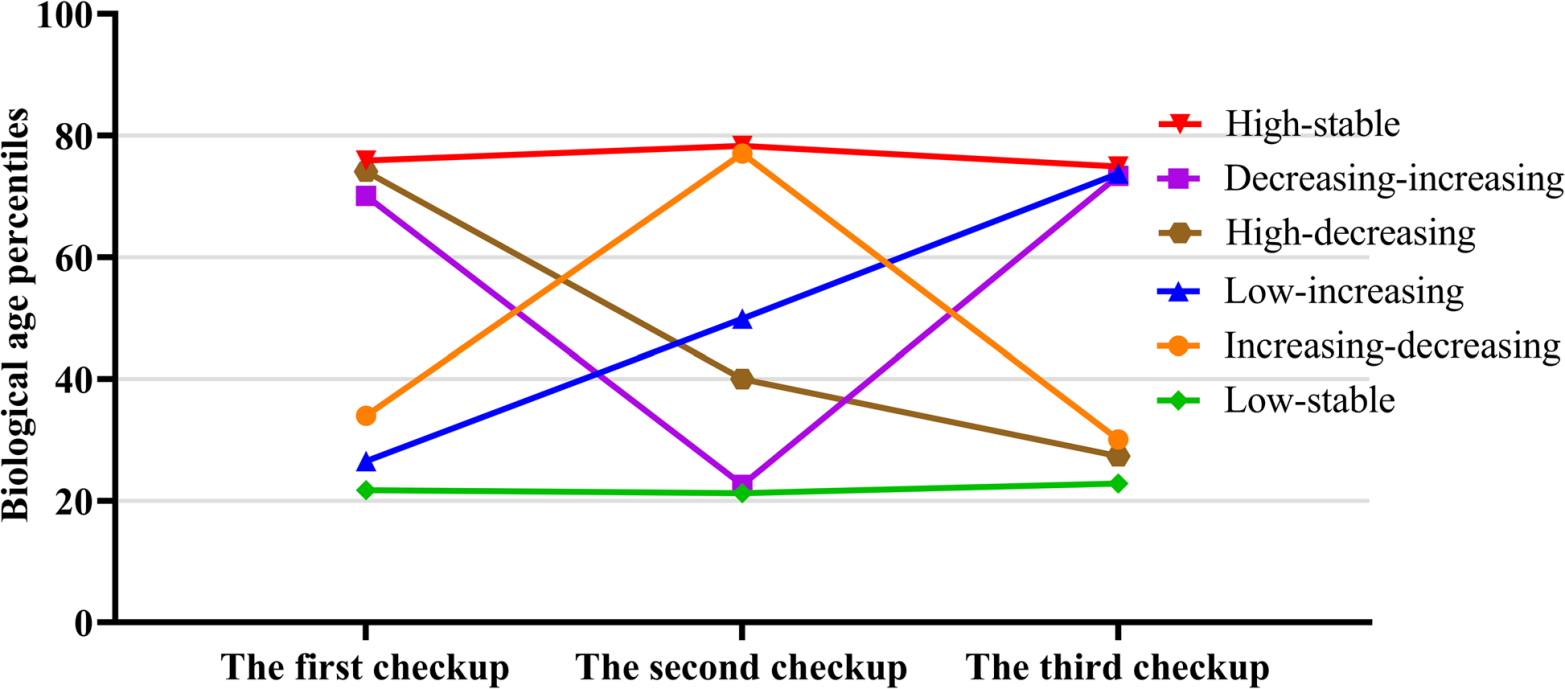
Mean biological age percentiles in the first, second, and third checkup, according to six aging trajectory patterns. Low-stable refers to a persistent low level aging state; Increasing–decreasing denotes an aging pattern that begins with low level aging status, followed by an upward and downward trajectory; Low-increasing indicates an aging trajectory beginning with low aging status and then continually increasing; High-decreasing denotes an aging trajectory beginning with a high degree of aging status, followed by persistent decline; Decreasing-increasing signifies an aging trajectory beginning with a high degree of aging status, followed by decline and then rise again; High-stable implies maintaining a persistently high state of aging trajectory. The number of participants is 40 071. Biological age was predicted by a biological age model constructed using the Deep Neural Networks (DNN) method with 32 clinical and biochemical indicators. CVD indicates cardiovascular disease

Aging trajectories were constructed for 40 071 participants, revealing six distinct trajectories based on BA percentiles over time (Fig. 3): low-stable (9728 [24.28%]), increasing-decreasing (4379 [10.93%]), low-increasing (5957 [14.87%]), high-decreasing (5858 [14.62%]), decreasing-increasing (3033 [7.57%]), and high-stable (11 116 [27.74%]). No significant difference in CA in the third checkup was observed among aging trajectories, and the low-stable group had the lowest BA while the high-stable had the highest (Table S1).

Further analysis showed that, in the low-increasing group, 13 indicators showed a decreasing trend over checkup time, while 19 indicators showed an increasing trend. 8 indicators exhibited significant differences in changes among individuals, while the rest showed no difference. In the high-decreasing group, 13 indicators showed a decreasing trend, 4 indicators remained unchanged, and 15 indicators showed an increasing trend. 9 indicators exhibited differences in changes among individuals, while the rest showed no significant difference. Between the two groups, there were statistically significant differences in the trends of 28 indicators: 17 indicators had consistent directions of change but different slopes, while 11 indicators had opposite directions of change. The specific changes in indicators are detailed in Table S2.

### The associations of baseline aging status with CVD and mortality

During 2006–2021, 6922 participants developed CVD events (median follow-up 14.98 years), and 10 317 deaths occurred (median follow-up 15.01 years). CVD subtypes comprised 1510 myocardial infarctions, 812 hemorrhagic strokes, and 4990 ischemic strokes, with 383 cases involving at least two subtypes.

**Fig. 4:**
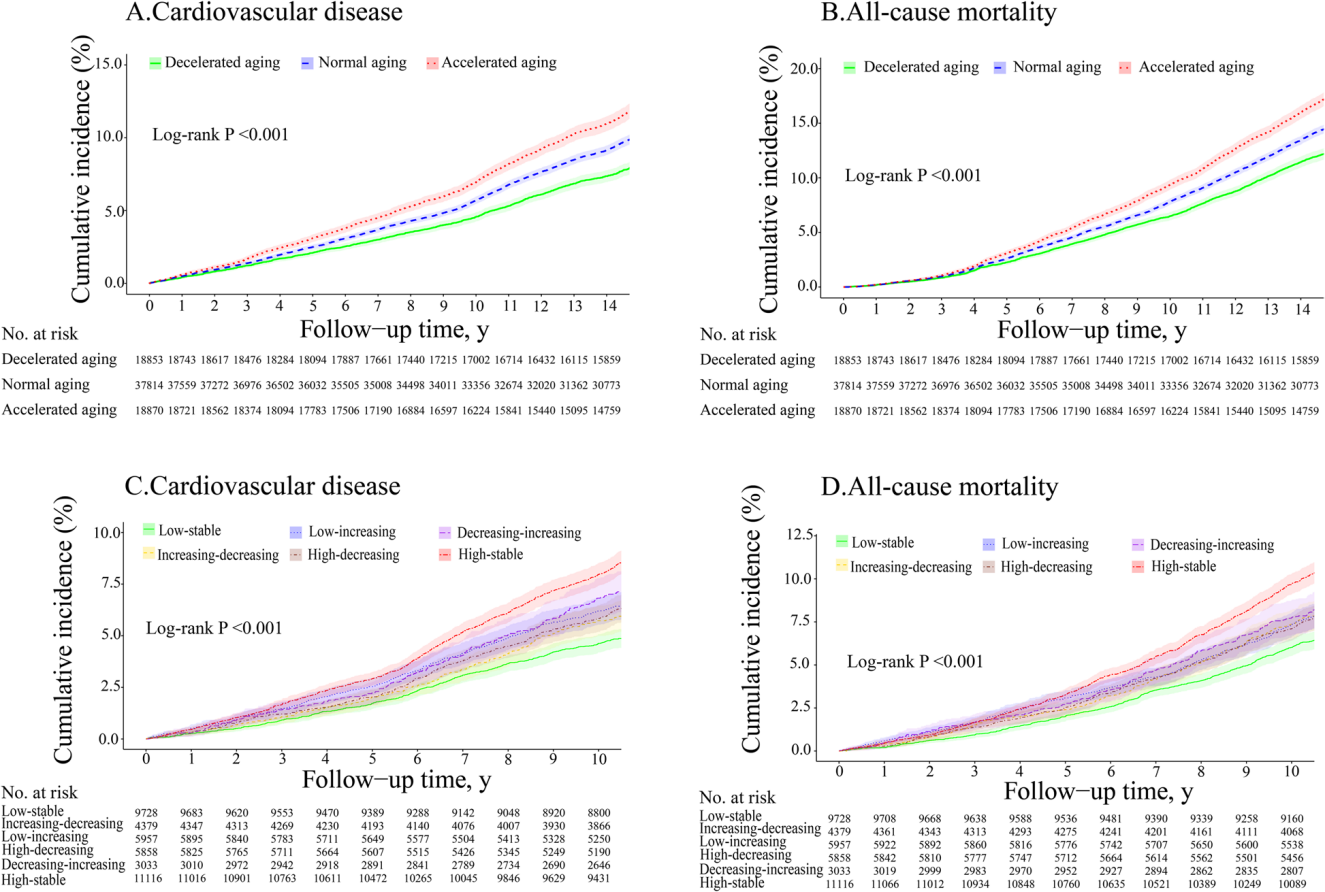
Kaplan–Meier estimates of the cumulative incidence of cardiovascular disease and all-cause death. **A-B** Kaplan–Meier curve of observed cumulative incidence of cardiovascular disease (**A**) and all-cause death (**B**) during 15 years of follow-up according to baseline aging status. **C-D** Kaplan–Meier curve of observed cumulative incidence of cardiovascular disease (**C**) and all-cause death (**D**) during 11 years of follow-up according to aging trajectory groups; Low-stable refers to a persistent low level aging state; Increasing–decreasing denotes an aging pattern that begins with low level aging status, followed by an upward and downward trajectory; Low-increasing indicates an aging trajectory beginning with low aging status and then continually increasing; High-decreasing denotes an aging trajectory beginning with a high degree of aging status, followed by persistent decline; Decreasing-increasing signifies an aging trajectory beginning with a high degree of aging status, followed by decline and then rise again; High-stable implies maintaining a persistently high state of aging trajectory

Kaplan–Meier curves showed significant differences (log‐rank test, *P* < 0.0001) in CVD risk among baseline aging statuses (Fig. 4, part A). Accelerated aging had the highest CVD incidence (7.99 per 1000 person-years), while decelerated aging had the lowest (5.44 per 1000 person-years). Compared with participants with normal aging, the *HRs* of Model 3 for CVD events were 1.17 (95% *CI* 1.11–1.23) for accelerated aging participants and 0.85 (0.80–0.90) for decelerated aging participants (Table 2). In the analyses of CVD subtypes, similar results were yielded for myocardial infarction and ischemic stroke, while decelerated aging did not significantly differ from normal aging in hemorrhagic stroke risk (Table 2).

**Table 2:** Association of baseline aging status with the risk of cardiovascular disease, cardiovascular disease subtypes, and all-cause mortality

Outcome	Baseline aging status	Cases/incident rate^a^	HR (95% CI) of Model 1	HR (95% CI) of Model 2	HR (95% CI) of Model 3
CVD	Decelerated aging	1424/5.44	0.81 (0.76–0.86)	0.81 (0.76–0.86)	0.85 (0.80–0.90)
	Normal aging	3469/6.70	Ref	Ref	Ref
	Accelerated aging	2029/7.99	1.20 (1.13–1.26)	1.20 (1.13–1.26)	1.17 (1.11–1.23)
CVD subtype—Myocardial infarction	Decelerated aging	295/1.10	0.76 (0.67–0.87)	0.76 (0.66–0.87)	0.80 (0.70–0.92)
	Normal aging	767/1.44	Ref	Ref	Ref
	Accelerated aging	448/1.71	1.19 (1.05–1.33)	1.19 (1.06–1.34)	1.16 (1.03–1.30)
CVD subtype—Hemorrhagic stroke	Decelerated aging	183/0.68	0.94 (0.79–1.13)	0.95 (0.80–1.13)	1.00 (0.84–1.19)
	Normal aging	386/0.72	Ref	Ref	Ref
	Accelerated aging	243/0.92	1.27 (1.08–1.50)	1.28 (1.09–1.50)	1.23 (1.05–1.45)
CVD subtype—Ischemic stroke	Decelerated aging	1028/3.88	0.81 (0.75–0.87)	0.81 (0.76–0.87)	0.85 (0.79–0.91)
	Normal aging	2506/4.78	Ref	Ref	Ref
	Accelerated aging	1456/5.65	1.19 (1.11–1.27)	1.18 (1.11–1.26)	1.16 (1.09–1.24)
All-cause mortality	Decelerated aging	2170/8.05	0.84 (0.80–0.88)	0.84 (0.80–0.88)	0.86 (0.82–0.90)
	Normal aging	5145/9.59	Ref	Ref	Ref
	Accelerated aging	3002/11.32	1.18 (1.13–1.23)	1.18 (1.13–1.23)	1.17 (1.12–1.22)

Model 1 adjusted for chronological age and sex. Model 2 additionally adjusted for education level, occupation, physical activity, smoking status, alcohol consumption, and salt consumption habit. Model 3 further adjusted for history of hypertension, history of diabetes, history of dyslipidemia, antihypertensive drug, antidiabetic drug, and lipid-lowering drug

*CI* Indicates confidence interval, *CVD* Cardiovascular disease, *HR* Hazard ratio, and *Ref* Reference

^a^Incident rate indicates per 1000 person-years

Similarly, baseline aging status was associated with mortality (Fig. 4, part B). Compared with normal aging, accelerated aging was a risk factor for mortality (*HR* of Model 3 was 1.17 [95% *CI* 1.12–1.22]), while participants with decelerated aging had a lower risk (*HR* of Model 3 was 0.86 [95% *CI* 0.82–0.90]) (Table 2).

### The associations of aging trajectory with CVD and mortality

During 2010–2021, 2680 CVD events (median follow-up 11.02 years) and 3234 deaths (median follow-up 11.05 years) were identified. CVD subtypes comprised 537 myocardial infarctions, 235 hemorrhagic strokes, and 2022 ischemic strokes, with 113 cases involving at least two subtypes.

**Fig. 5:**
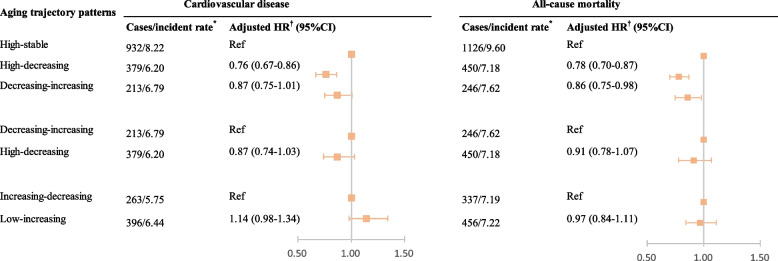
Association of aging trajectory with the risks of cardiovascular disease and all-cause mortality: comparison of different trajectory groups. ^*^Incident rate indicates per 1000 person-years. ^**†**^Adjusted for chronological age, sex, education level, occupation, physical activity, smoking status, alcohol consumption, salt consumption habit, self-reported hypertension history, self-reported diabetes history, self-reported dyslipidemia history, antihypertensive drug, antidiabetic drug, and lipid-lowering drug. High-stable implies maintaining a persistently high state of aging trajectory; High-decreasing denotes an aging trajectory beginning with a high degree of aging status, followed by persistent decline; Decreasing-increasing signifies an aging trajectory beginning with a high degree of aging status, followed by decline and then rise again; Increasing–decreasing denotes an aging pattern that begins with low level aging status, followed by an upward and downward trajectory; Low-increasing indicates an aging trajectory beginning with low aging status and then continually increasing. CI indicates confidence interval; and HR, hazard ratio

Kaplan–Meier curves showed that participants in the high-stable group had the highest CVD risk, and the lowest risk was observed in the low-stable group (Fig. 4, part C). Compared with participants in the low-stable group, participants in other groups, except those in the increasing-decreasing group, had higher CVD risk (Table 3). The high-stable group had the highest *HR* (*HR* of Model 3 was 1.62 [95% *CI* 1.45–1.81]), followed by the decreasing-increasing group (*HR* of Model 3 was 1.41 [1.20–1.65]) and low-increasing group (*HR* of Model 3 was 1.32 [1.16–1.51]). Conversely, compared to the high-stable group, participants in the high-decreasing group had 24% lower CVD risk but no statistically significant difference in the decreasing-increasing group (Fig. 5). No statistically significant difference in CVD risk was observed among other group comparisons (Fig. 5).

**Table 3:** Association of aging trajectory with the risks of cardiovascular disease, cardiovascular disease subtypes, and all-cause mortality

Outcome	Aging trajectory groups^a^	Cases/incident rate^b^		HR (95% CI) of Model 1	HR (95% CI) of Model 2		HR (95% CI) of Model 3
CVD	Low-stable	497/4.85		Ref	Ref		Ref
	Increasing-decreasing	263/5.75		1.21 (1.04–1.40)	1.19 (1.02–1.38)		1.16 (1.00–1.35)
	Low-increasing	396/6.44		1.34 (1.17–1.53)	1.35 (1.18–1.54)		1.32 (1.16–1.51)
	High-decreasing	379/6.2		1.28 (1.12–1.46)	1.26 (1.10–1.44)		1.23 (1.07–1.40)
	Decreasing-increasing	213/6.79		1.46 (1.24–1.71)	1.44 (1.23–1.70)		1.41 (1.20–1.65)
	High-stable	932/8.22		1.72 (1.54–1.92)	1.70 (1.53–1.90)		1.62 (1.45–1.81)
CVD subtype—Myocardial infarction	Low-stable	105/1.01		Ref	Ref		Ref
	Increasing-decreasing	49/1.05		1.06 (0.75–1.48)	1.06 (0.75–1.48)		1.03 (0.73–1.45)
	Low-increasing	77/1.23		1.22 (0.91–1.63)	1.23 (0.92–1.66)		1.21 (0.90–1.62)
	High-decreasing	62/0.99		0.98 (0.72–1.34)	0.99 (0.72–1.35)		0.96 (0.70–1.31)
	Decreasing-increasing	45/1.40		1.44 (1.02–2.04)	1.46 (1.03–2.08)		1.43 (1.01–2.03)
	High-stable	199/1.71		1.71 (1.35–2.16)	1.72 (1.36–2.18)		1.63 (1.28–2.07)
CVD subtype—Hemorrhagic stroke	Low-stable	40/0.38		Ref	Ref		Ref
	Increasing-decreasing	23/0.49		1.30 (0.78–2.17)	1.28 (0.77–2.14)		1.27 (0.76–2.12)
	Low-increasing	32/0.51		1.32 (0.83–2.10)	1.30 (0.82–2.07)		1.28 (0.80–2.04)
	High-decreasing	34/0.54		1.41 (0.89–2.23)	1.40 (0.89–2.21)		1.37 (0.87–2.17)
	Decreasing-increasing	21/0.65		1.75 (1.03–2.97)	1.72 (1.01–2.92)		1.68 (0.99–2.86)
	High-stable	85/0.73		1.91 (1.31–2.78)	1.84 (1.26–2.68)		1.76 (1.20–2.57)
CVD subtype—Ischemic stroke	Low-stable	367/3.56		Ref	Ref		Ref
	Increasing-decreasing	199/4.32		1.23 (1.04–1.46)	1.21 (1.02–1.43)		1.18 (0.99–1.40)
	Low-increasing	307/4.96		1.41 (1.21–1.64)	1.42 (1.22–1.65)		1.38 (1.19–1.61)
	High-decreasing	296/4.81		1.35 (1.16–1.57)	1.33 (1.14–1.55)		1.29 (1.11–1.51)
	Decreasing-increasing	159/5.02		1.46 (1.22–1.76)	1.44 (1.20–1.74)		1.41 (1.17–1.70)
	High-stable	694/6.07	1.73 (1.52–1.96)			1.70 (1.50–1.94)	1.62 (1.43–1.84)
All-cause mortality	Low-stable	619/5.92	Ref			Ref	Ref
	Increasing-decreasing	337/7.19	1.26 (1.10–1.43)			1.24 (1.09–1.42)	1.22 (1.07–1.39)
	Low-increasing	456/7.22	1.22 (1.08–1.38)			1.22 (1.09–1.38)	1.20 (1.06–1.35)
	High-decreasing	450/7.18	1.24 (1.10–1.40)			1.24 (1.10–1.40)	1.22 (1.08–1.37)
	Decreasing-increasing	246/7.62	1.35 (1.16–1.56)			1.34 (1.16–1.56)	1.33 (1.14–1.54)
	High-stable	1126/9.60	1.64 (1.49–1.81)			1.62 (1.47–1.79)	1.55 (1.41–1.71)

Model 1 adjusted for chronological age and sex. Model 2 additionally adjusted for education level, occupation, physical activity, smoking status, alcohol consumption, and salt consumption habit. Model 3 further adjusted for history of hypertension, history of diabetes, history of dyslipidemia, antihypertensive drug, antidiabetic drug, and lipid-lowering drug

*CI* Indicates confidence interval, *CVD* Cardiovascular disease, *HR* Hazard ratio, and *Ref* Reference

^a^Low-stable refers to a persistent low level aging state; Increasing–decreasing denotes an aging pattern that begins with low level aging status, followed by an upward and downward trajectory; Low-increasing indicates an aging trajectory beginning with low aging status and then continually increasing; High-decreasing denotes an aging trajectory beginning with a high degree of aging status, followed by persistent decline; Decreasing-increasing signifies an aging trajectory beginning with a high degree of aging status, followed by decline and then rise again; High-stable implies maintaining a persistently high state of aging trajectory

^b^Incident rate indicates per 1000 person-years

The Cox model results of ischemic stroke were similar to the overall CVD events. Relative to the low-stable group, the high-stable group and the decreasing-increasing group had a higher myocardial infarction risk; the high stable group also had a higher hemorrhagic stroke risk. (Table 3).

Aging trajectories were associated with mortality (Fig. 4, part D). Compared with the low-stable group, participants in other groups had a higher mortality (Table 3). Additionally, compared with the high-stable group, the high-decreasing group and the decreasing-increasing group had decreased mortality, with *HRs* of 0.78 (95% CI 0.70–0.87) and 0.86 (0.75–0.98), respectively (Fig. 5). No statistically significant difference in mortality was observed among other group comparisons (Fig. 5).

### Sensitivity analyses

Sensitivity analyses confirmed the primary findings regarding the associations of baseline aging status and aging trajectory with CVD and mortality (Tables S3 and S4). Specifically, when excluding participants with a self-reported disease history and medication usage, the increasing–decreasing group displayed heightened CVD risk compared to the low-stable group. Reconstructed aging trajectories (excluding participants with missing BA at any checkup) yielded six similar trajectories (Fig. S3) and maintained their associations with incident CVD and mortality (Table S4).

## Discussion

In this large prospective cohort study, compared to those with normal aging, participants with accelerated aging had higher risks of CVD and death, and those with decelerated aging had lower risks. This finding held for myocardial infarction and ischemic stroke but not hemorrhagic stroke. Six distinct aging trajectories were identified. Compared with the low-stable group, participants in other aging trajectories had higher risks of CVD (except for the increasing-decreasing group) and death. Compared with the high-stable group, the high-decreasing group showed lower risks of both CVD and death, and the decreasing-increasing group also had lower death risk. Additionally, the association of aging trajectories with ischemic stroke was like the overall CVD events, but this similarity did not extend to myocardial infarction or hemorrhagic stroke.

There have been studies using varying numbers of clinical indicators and methods to construct BA models. Some studies predict BA based on over a dozen indicators using methods like KDM or Levine, achieving correlations with CA above 0.9, primarily because the prediction models include CA as an indicator [13, 17]. Others use machine learning methods such as LightGBM, extreme gradient boosting, and support vector machine based on 48 to 78 indicators, reaching correlations with CA above 0.7 [20, 33, 34]. We predict BA using 32 indicators and the DNN method, with a correlation slightly lower than studies using 42 indicators [16] but comparable to those using 20 indicators in a Korean population [23]. The differences in DNN model prediction performance may be due to variations in population ethnicity and the indicators used. Notably, the absence of total protein, albumin, and direct bilirubin might be a primary reason (when data from the third examination including these three indicators is used, the correlation can increase to above 0.73, data not shown). Despite this, our model still demonstrates a certain level of predictive capability.

Previous studies employed traditional methods (e.g., KDM, LightGBM, Gompertz proportional hazard models) to develop BA and aging index, which established their correlation with CVD and mortality in some cohorts like the fourth National Health and Nutrition Examination Survey (NHANES IV) [17], UK biobank [15, 20], and the China Kadoorie Biobank (CKB) [13]. Putin et al. [16] pioneered a BA using the DNN model from blood routine and biochemistry tests, verifying its accuracy across diverse populations and its predictive power for mortality [23]. However, whether such predictive ability extends to CVD has not been reported. Our study is the first to show that DNN-derived BA, like its traditionally derived counterpart, can predict CVD risk and specific CVD subtypes.

The association between BA and mortality, as shown in our study, is consistent with findings from multiple cohorts, including the UK biobank [13, 15, 20], NHANES IV [17], and several Chinese cohorts [13, 35]. It is also consistent with studies using a frailty index constructed by assigning weight or score [18, 36–38]. A Swedish study even demonstrated that BA, as indicated by the presence of cognitive impairment or physical limitation, plays a pivotal role in the associations of blood pressure with cardiovascular and non-cardiovascular mortality in older adults [39]. These findings indicate that research on the impact of BA on mortality is indeed extensive and profound. However, investigations into the association between BA and CVD, particularly its subtypes, remain insufficient. Liu et al. [20] discovered in the UK biobank population (37–73 years) that accelerated aging is a risk factor for all CVD subtypes except hemorrhagic stroke, while participants with decelerated aging exhibited a lower risk for all CVD subtypes. Another study in the same cohort also showed a higher frailty index relative to increased major vascular events [40]. The American Cardiovascular Health Study, involving participants aged ≥ 65, showed that the aging index provided model discrimination of risk for composite outcomes (CVD, death, cancer, etc.) [18]. These findings were made in specific age groups within European and American populations. Our research in the Chinese population aged ≥ 18 echoes these findings, suggesting that our constructed aging status can be employed in primary healthcare to categorize individuals across different CA.

The association between aging status and hemorrhagic stroke differs from UK biobank findings [20]. The primary pathoetiologies of hemorrhagic stroke are chronic hypertension, cerebral amyloid angiopathy, and some hereditary vascular diseases [41]. Even though the BA in our study incorporated limited risk factors (e.g., blood pressure), it showed that, compared with normal aging, accelerated aging was associated with an increased risk of hemorrhagic stroke, whereas no significant correlation between decelerated aging and a lower risk was observed. Inconsistent findings with the UK biobank may result from differences in hemorrhagic stroke coverage, the indicators and methods constructed for BA, and participant demographics.

We identified six heterogeneous aging trajectories and assessed their associations with CVD and mortality. In a prior cohort study of individuals aged ≥ 50 years, three homogenous aging trajectories were identified, with medium/high‑degree accelerated aging trajectory demonstrating elevated mortality risks versus the slow aging trajectory [22]. Several studies employed frailty scale scores to discern multiple trajectories in the elderly population, revealing increased healthcare utilization and inferior survival outcomes among individuals with consistently elevated frailty levels [42–44]. This study pioneers the identification of six heterogeneous aging trajectories in adults ≥ 18 years, revealing that individuals with persistently low aging trajectory (low-stable) had the lowest CVD and mortality risks, whereas other trajectories conferred increased risk, peaking in those with consistently high aging trajectory (high-stable). Our findings underscore the benefits of maintaining a persistently low-stable aging level. Individuals initially with high aging levels but declining then (high-decreasing) exhibited lower CVD risk and mortality than those in the high-stable group. Those in a decreasing-increasing group showed reduced mortality only. It suggests that individuals with high aging levels can mitigate CVD risk and mortality through interventions to decrease their aging status, necessitating long-term persistence for optimal outcomes. Conversely, those starting at low aging levels see increased CVD and death risks if their status is not sustained.

For both the low-increasing and high-decreasing groups, this study provides a detailed examination of the trends in the 32 indicators over the initial three checkups. It clarifies that in the low-increasing group, there are bidirectional changes in the indicators—some decreasing while others increasing—and several of these indicators exhibit significant variation among individuals. In the high-decreasing group, indicators show decreases, stability, and increases, with some also showing significant differences among individuals. Moreover, the differences in the trends of these indicators between the two groups are clearly demonstrated, particularly highlighting that indicators with opposite directions of change could become important targets for future intervention studies.

This large and long-term prospective cohort study has some advantages and public health significance. First, our study is the first to correlate DNN BA-based aging status with CVD risk, capitalizing on our large sample to explore CVD subtypes. The results showed that this aging status can not only predict mortality but also CVD risks. Notably, BA is a complex composite evaluation metric based on 32 common clinical indicators. A change in a single indicator may influence others, thereby impacting the overall aging status. Therefore, we recommend that individuals should focus on indicators that deviate from population-normal levels when aiming to prevent CVD and reduce mortality. They should adopt a holistic health management strategy rather than targeting only the highest-risk factors. However, the feasibility and effectiveness of personalized intervention guidance and health management in practical applications require further research for assessment. Second, most studies have only investigated BA or aging index measured at a single point, which might not completely capture the longitudinal changes in the risk of CVD and death. Single measurements fail to account for the dynamic nature of aging and its impact on outcomes over time. Therefore, it is crucial to consider the long-term patterns of aging when assessing adverse outcome risk in individuals. The longitudinal design of our study allowed for repeated measurements of the variables of interest, enabling us to account for intraindividual changes over time. We uncovered six distinct aging trajectories, highlighting the importance of maintaining persistently low aging levels. Trajectory modeling provides valuable insights into the intricate relationships between aging and the risk of CVD and death. In addition, the study’s representativeness is enhanced by including adults of all ages ≥ 18.

Our study has limitations. First, the findings from the Kailuan cohort, as a single-center prospective study population, should be cautiously extrapolated to other populations. Second, given that the vast majority of participants are male, it is important to recognize that CVD risks differ between sexes [45]. To mitigate the effects of sex disparities to the greatest extent, our BA model, percentile calculations, and classifications of aging status classification were all tailored specifically to each sex. Nonetheless, in light of the relatively small number of outcome incidents, we opted to adjust for sex within the Cox proportional hazards model as a covariate, rather than conducting separate, gender-specific analyses. Third, missing data inherent in large-scale, longitudinal checkup cohorts with repeated measurement may introduce information bias in the aging trajectory; however, sensitivity analyses confirmed the robust nature of the findings. Finally, the BA model is constructed based on 32 indicators, selected for their data completeness, similar to many previous studies [16, 23, 33, 34]. Notably, some variables potentially covary with each other. Although the DNN algorithm can mitigate the negative impacts of collinearity on the model, issues such as multicollinearity and model overfitting still require attention. In future research aimed at optimizing the BA model, it may be beneficial to select the most predictive indicators from each category and fit them in a way that minimizes collinearity among indicator groups.

## Conclusions

Aging status and aging trajectory derived by DNN-BA were associated with CVD and mortality. Compared with normal aging, accelerated aging was associated with a higher risk of CVD and mortality, while decelerated aging was associated with a lower risk. Individuals with persistently low aging levels exhibited the lowest CVD risk and mortality, whereas those with constantly high aging levels bore the highest risk. Those whose aging levels were initially high and subsequently declined showed reduced CVD and death risks compared to those with consistently high aging levels. These results indicate that aging status holds potential as a useful risk stratification tool in primary healthcare. We emphasize that maintaining consistently low aging levels can mitigate CVD and death risks, even for those initially classified as having high aging status. It is important to note that further research is needed to validate these results across different populations.

## Supplementary Information

Supplementary Material 1.

Supplementary Material 2.

Supplementary Material 3.

## Data Availability

The datasets used and/or analyzed during the current study are available from the corresponding author on reasonable request.

## Abbreviations

BIC: Bayesian Information Criterion
BA: Biological age
CA: Chronological age
CKB: China Kadoorie Biobank
CVD: Cardiovascular diseases
DNN: Deep neural networks
KDM: Klemera Doubal method
NHANES IV: The fourth National Health and Nutrition Examination Survey
