# Supplementary material for: Association of clinical biomarker-based biological age and aging trajectory with cardiovascular disease and all-cause mortality in Chinese adults: a population-based cohort study
Source: BMC Public Health. 2025 Mar 4;25:868. doi: 10.1186/s12889-025-22114-7 (PMC11881332; doi:10.1186/s12889-025-22114-7)
Supplement: Supplementary file 1 — Supplementary Material 1. [file 12889_2025_22114_MOESM1_ESM.docx]

**Supplemental Material: Figures S1-S3**

**
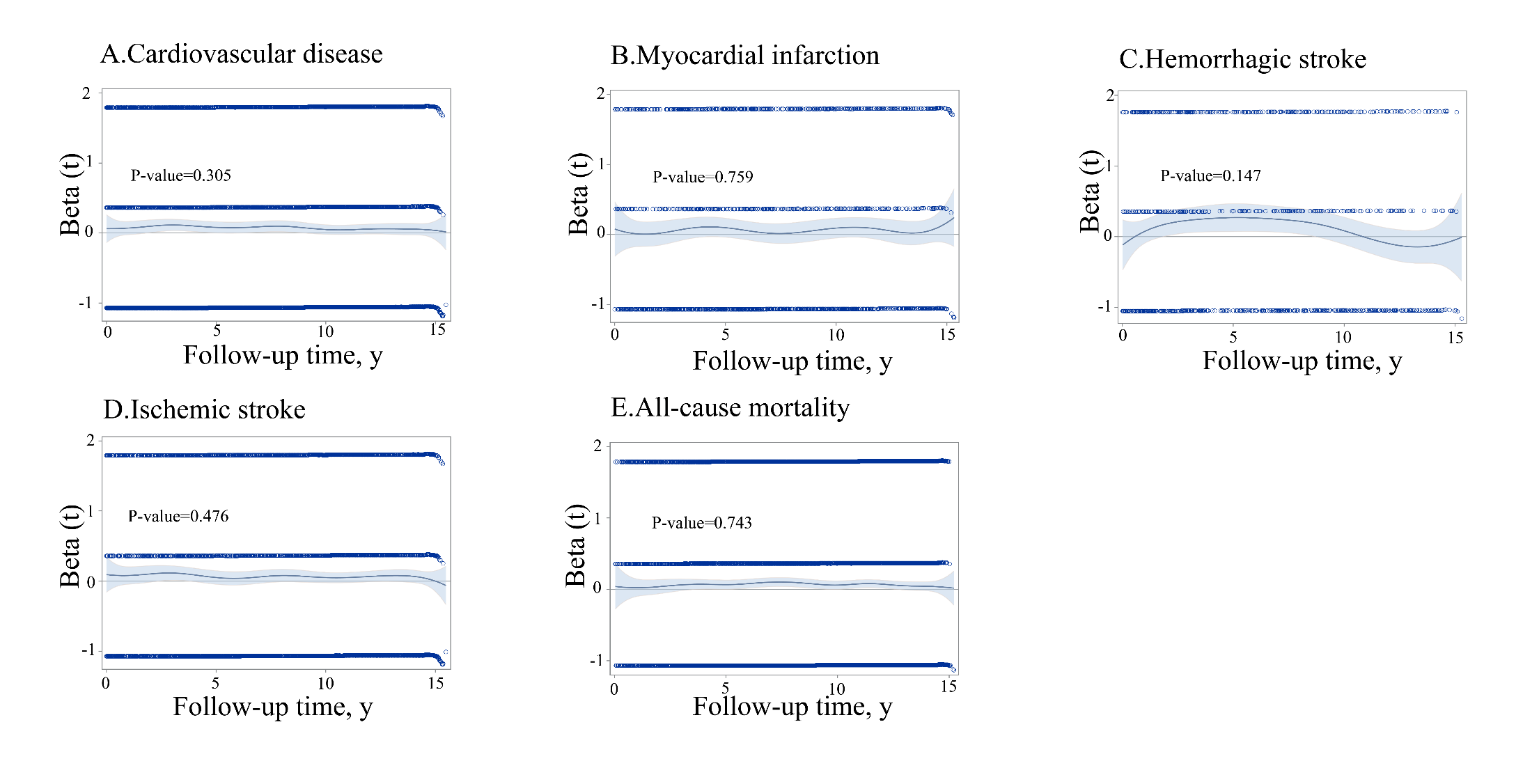
**

**Figure S1. The scaled Schoenfeld residual plots for the baseline aging status were generated using Cox models that examined the associations of baseline aging status with CVD, CVD subtypes, and all-cause mortality.**

The P-values on the plots represent the global test of the Schoenfeld residuals, indicating all covariates as a whole satisfy the proportional hazards assumption in the Cox models.

CVD indicates cardiovascular disease.

**
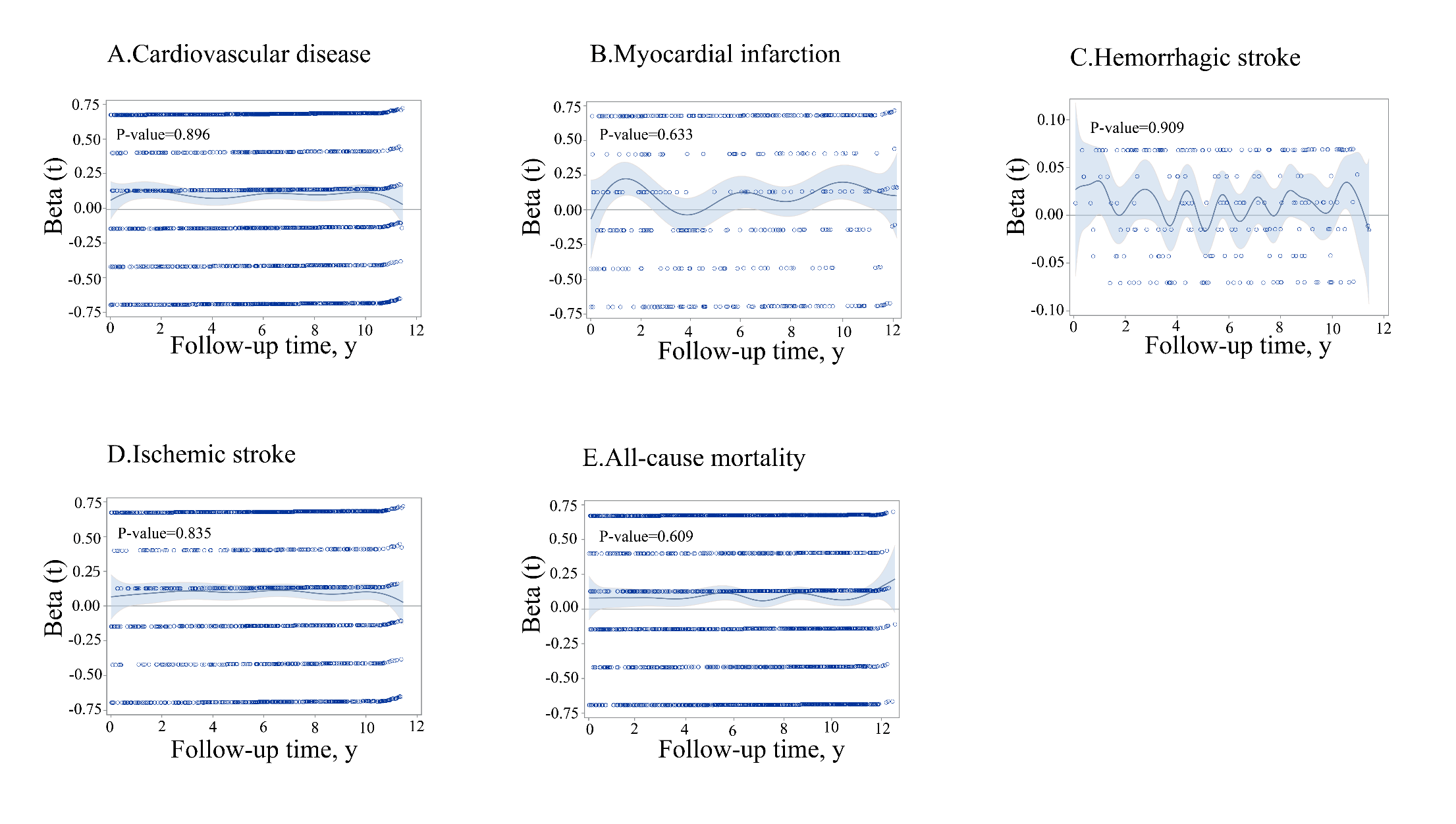
**

**Figure S2. The scaled Schoenfeld residual plots for the aging trajectories were generated using Cox models that examined the associations of aging trajectories with CVD, CVD subtypes, and all-cause mortality.**

The P-values on the plots represent the global test of the Schoenfeld residuals, indicating all covariates as a whole satisfy the proportional hazards assumption in the Cox models.

CVD indicates cardiovascular disease.

**
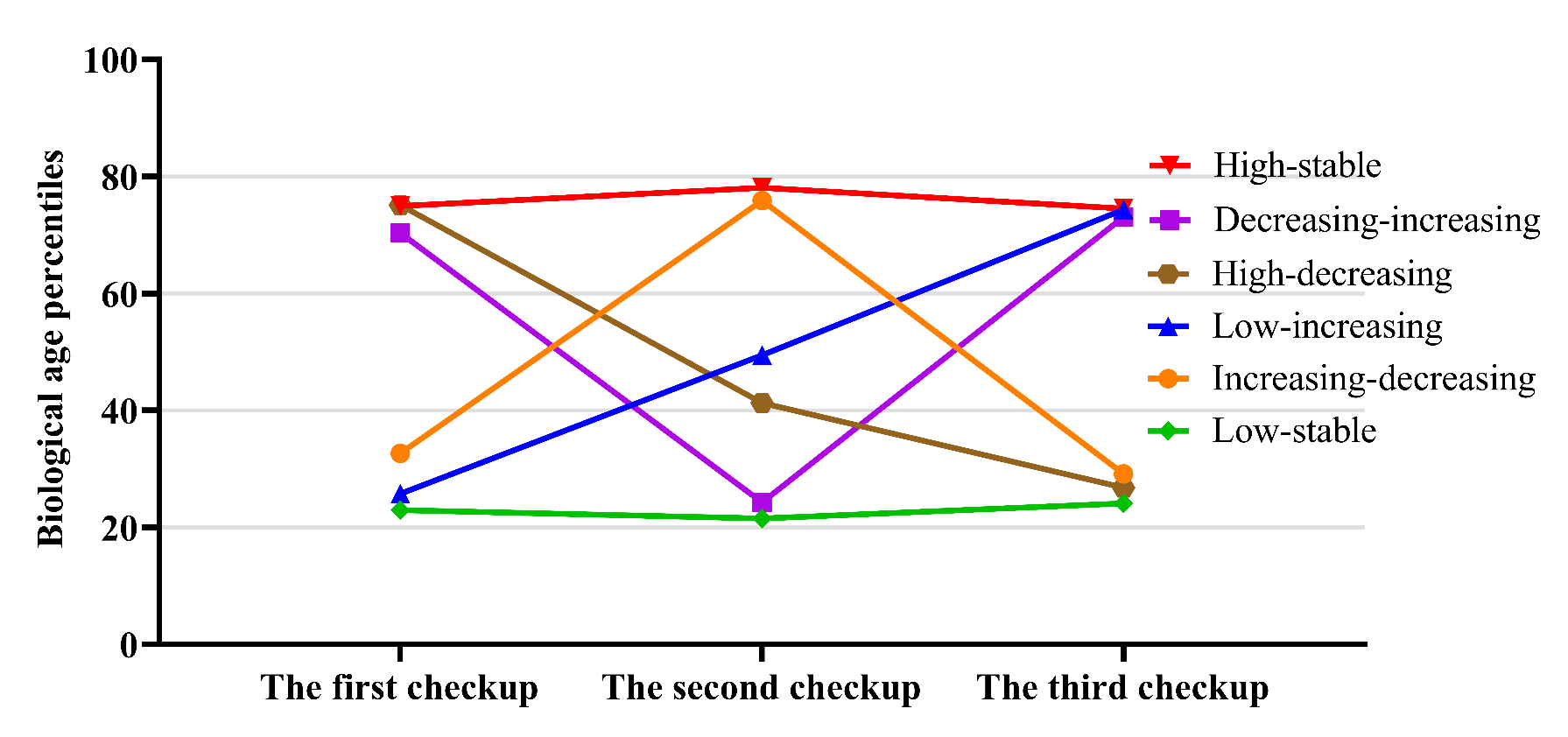
**

**Figure S3. Mean biological age percentiles in the first, second, and third checkup, according to six aging trajectory patterns: excluding participants with missing biological age at any checkup.**

Low-stable refers to a persistent low level aging state; Increasing-decreasing denotes an aging pattern that begins with low level aging status, followed by an upward and downward trajectory; Low-increasing indicates an aging trajectory beginning with low aging status and then continually increasing; High-decreasing denotes an aging trajectory beginning with a high degree of aging status, followed by persistent decline; Decreasing-increasing signifies an aging trajectory beginning with a high degree of aging status, followed by decline and then rise again; High-stable implies maintaining a persistently high state of aging trajectory.

The number of participants is 27 967.

Biological age was predicted by a biological age model constructed using the Deep Neural Networks (DNN) method with 32 clinical and biochemical indicators. CVD indicates cardiovascular disease.
