# Supplementary material for: Association of clinical biomarker-based biological age and aging trajectory with cardiovascular disease and all-cause mortality in Chinese adults: a population-based cohort study
Source: BMC Public Health. 2025 Mar 4;25:868. doi: 10.1186/s12889-025-22114-7 (PMC11881332; doi:10.1186/s12889-025-22114-7)
Supplement: Supplementary file 2 — Supplementary Material 2. [file 12889_2025_22114_MOESM2_ESM.docx]

**Supplemental Methods: Procedures and results of Biological Age models construction**

1. Dataset and data preprocessing

The first, second, and third checkup data in the Kailuan study were used to construct three biological age (BA) models, respectively, and three datasets were generated. The number of participants in the three datasets was 101 510, 101 133, and 92 967, respectively.

Following the method of previous literature,^1-6^ we selected 36 indicators from the Kailuan study datasets as candidate features for the BA model, which were known to indicate the function, structure, and/or general health of the cardiovascular, hepatic, renal, immune, and metabolic systems. The percentile threshold method was used to identify extreme outliers for each indicator in three datasets. The upper threshold was 99.95th percentile, and the lower threshold was 0.05th percentile. The value more than the upper threshold or less than the lower threshold was transformed into a missing value. Then, we excluded indicators with high missing rates (heart rate with missing rate>15% was excluded). Total protein, albumin, and direct bilirubin were excluded because they were only available in the third checkup. Ultimately, we included 32 indicators to construct the BA models. Indicators are as follows:

- Cardiovascular indicators: systolic blood pressure (SBP) and diastolic blood pressure (DBP).
- Metabolic indicators: body mass index (BMI), waist to hip ratio (WHR), fasting blood glucose (FBG), total cholesterol (TC), triglyceride (TG), high density lipoprotein cholesterol (HDL-C), low density lipoprotein cholesterol (LDL-C).
- Hepatic indicators: alanine aminotransferase (ALT), total bilirubin (TBIL) in blood.
- Renal indicators: creatinine (Cr), blood urea nitrogen (BUN), uric acid (UA).
- Immune indicators: hypersensitive C-reactive protein (hs-CRP) and blood hematology tests of leukocytes, erythrocytes, thrombocytes, and hemoglobin.

Furthermore, participants with missing values for any of the 32 indicators were excluded, resulting in a final dataset consisting of 78 367 individuals for the first checkup (chronological age range: 18.23-95.62 years for males, 18.10-87.77 for females), 74 061 individuals for the second checkup (chronological age range: 18.11-98.92 years for males, 19.19-92.70 for females), and 75 917 for the third checkup (chronological age range: 18.02-98.85 years for males, 18.93-91.60 for females). The characteristics of indicators in each checkup dataset are shown in **Table**.

**Table. Characteristics of indicators in each checkup dataset**

| **Indicators** | **The first checkup**  **(2006-2007)** | **The second checkup**  **(2008-2009)** | **The third checkup**  **(2010-2011)** |
| --- | --- | --- | --- |
| The number of participants | 78 367 | 74 061 | 75 917 |
| Chronological age, y | 51.48 (12.39) | 51.37 (12.37) | 51.52 (13.00) |
| Sex, n (%) |  |  |  |
| Female | 15 978 (20.40) | 14 552 (19.60) | 15 899 (20.90) |
| Male | 62 389 (79.60) | 59 509 (80.40) | 60 018 (79.10) |
| SBP, mmHg | 130.98 (20.51) | 131.20 (20.27) | 130.28 (19.31) |
| DBP, mmHg | 83.63 (11.53) | 84.57 (11.48) | 84.18 (10.89) |
| BMI, kg/m^2^ | 25.07 (3.45) | 24.91 (3.39) | 25.07 (3.39) |
| WHR | 0.89 (0.07) | 0.90 (0.07) | 0.90 (0.06) |
| FBG, mmol/L | 5.46 (1.65) | 5.65 (1.60) | 5.63 (1.49) |
| TC, mmol/L | 4.94 (1.14) | 5.00 (1.00) | 4.94 (0.98) |
| TG, mmol/L | 1.27 (0.90, 1.93) | 1.28 (0.87, 1.83) | 1.29 (0.92, 1.93) |
| HDL-C, mmol/L | 1.56 (0.40) | 1.48 (0.39) | 1.51 (0.43) |
| LDL-C, mmol/L | 2.38 (0.89) | 2.64 (0.83) | 2.59 (0.81) |
| ALT, U/L | 21.10 (15.52) | 21.29 (14.88) | 21.81 (16.54) |
| TBIL, μmol/L | 13.18 (5.65) | 13.41 (5.48) | 14.03 (5.71) |
| Cr, μmol/L | 91.90 (25.13) | 88.12 (23.99) | 81.80 (20.34) |
| BUN, mmol/L | 5.66 (1.53) | 5.53 (1.57) | 5.75 (1.55) |
| UA, μmol/L | 287.84 (82.42) | 280.88 (80.71) | 296.18 (88.48) |
| hs-CRP, median (IQR), mg/L | 0.79 (0.30, 2.07) | 1.58 (0.80, 3.20) | 1.06 (0.47, 2.53) |
| WBC, ×10^9^ /L | 6.57 (1.60) | 5.96 (1.53) | 6.40 (1.58) |
| NEUT, ×10^9^ /L | 3.91 (1.19) | 3.55 (1.13) | 3.89 (1.20) |
| LYM, ×10^9^ /L | 2.27 (0.65) | 2.08 (0.62) | 2.13 (0.62) |
| MON, median (IQR), ×10^9^ /L | 0.40 (0.30, 0.50) | 0.30 (0.20, 0.40) | 0.30 (0.21, 0.41) |
| NEUT% | 57.67 (7.92) | 57.42 (7.64) | 59.13 (7.79) |
| LYM% | 35.69 (7.40) | 36.13 (7.36) | 34.27 (7.38) |
| MON% | 6.62 (2.26) | 6.43 (2.25) | 6.19 (2.02) |
| RBC, ×10^12^/L | 4.93 (0.50) | 4.84 (0.53) | 4.85 (0.48) |
| HCT | 0.44 (0.05) | 0.44 (0.05) | 0.44 (0.04) |
| MCV, fL | 89.55 (6.50) | 90.54 (6.78) | 91.48 (5.52) |
| HGB, g/L | 149.78 (15.40) | 144.64 (15.36) | 148.85 (14.65) |
| MCH, pg | 30.47 (2.18) | 29.98 (2.21) | 30.77 (2.15) |
| MCHC, g/L | 340.89 (22.38) | 331.88 (22.92) | 336.71 (18.13) |
| PLT, ×10^9^ /L | 204.95 (54.86) | 199.66 (51.23) | 220.68 (55.72) |
| MPV, fL | 7.55 (0.83) | 7.62 (0.84) | 8.07 (1.26) |
| PCT, median (IQR) | 0.15 (0.12, 0.18) | 0.15 (0.12, 0.18) | 0.18 (0.14, 0.20) |
| PDW, % | 13.61 (1.85) | 13.27 (1.62) | 12.88 (1.89) |

Note: Data are mean (SD), except where otherwise stated. ALT = Alanine aminotransferase. BMI = Body mass index. BUN = Blood urea nitrogen. Cr = Creatinine. DBP = Diastolic blood pressure. FBG = Fasting blood glucose. HCT = Hematocrit. HDL-C = High density lipoprotein cholesterol. HGB = Hemoglobin. hs-CRP = Hypersensitive C-reactive protein. IQR = Interquartile range. LDL-C = Low density lipoprotein cholesterol. LYM = Lymphocyte. LYM% = The percentage of lymphocyte. MCH = Mean corpuscular hemoglobin. MCHC = Mean corpuscular hemoglobin concentration. MCV = Mean corpuscular volume. MON = Monocytes. MON% = The percentage of monocytes. MPV = Mean platelet volume. NEUT = Neutrophil count. NEUT% = The percentage of neutrophil. PCT = Platelet crit. PDW = Platelet distribution width. PLT = Platelet count. RBC = Red blood cell count. SBP = Systolic blood pressure. SD = Standard deviation. TBIL = Total bilirubin. TC = Total cholesterol. TG = Triglyceride. UA = Uric acid. WBC = White blood cell count. WHR = Waist-to-hip ratio.

2. Biological Age models

Deep Neural Networks (DNN) method was used to construct BA models, as previously reported.^1,2^ We used a DNN model with multiple layers, which allows fitting data with a high degree of dependencies between the input features (32 indicators) and the output feature (BA). The process of model construction is as follows:

(1) By dividing each checkup dataset into male and female datasets, we obtained six datasets, including the first checkup dataset (male), the first checkup dataset (female), the second checkup dataset (male), the second checkup dataset (female), the third checkup dataset (male), the third checkup dataset (female).

(2) For each dataset, 32 indicators were normalized between 0 and 1 by the “MinMaxScaler” method, respectively. The formula is

$X_{norm}=(X-X_{min})/(X_{max}-X_{min})$,

where $X$ is the original value of the indicator, $X_{max}$ is the maximum of the indicator, $X_{min}$ is the minimum of the indicator, and $X_{norm}$ is the transformed value that range is 0-1.

(3) Each dataset was randomly divided into a training set for fitting, a validation set for optimizing, and a testing set for evaluating with a ratio of 7:1:2.

(4) DNN models were used to construct BA models. For each model, a grid search over the space of model parameters was applied to find the best performing network architecture. The mean absolute error (MAE) loss function was used as an objective for neural networks. The hyperparameters of models were the activation function, learning rate, decay step, decay rate, hidden layers, optimizer, dropout probability, training batch size, and training step.

(5) The best-performing DNN models consisted of 3 hidden layers and 1 output layer (see Figure below). The output layer corresponds to the regression output. The models used a Clipped Rectified Linear Unit (Clipped ReLU) activation function and AdaGrad as the optimizer for the loss function. The dropout probability was set at 0.1 for datasets labeled as male and 0.3 for datasets labeled as female, respectively.


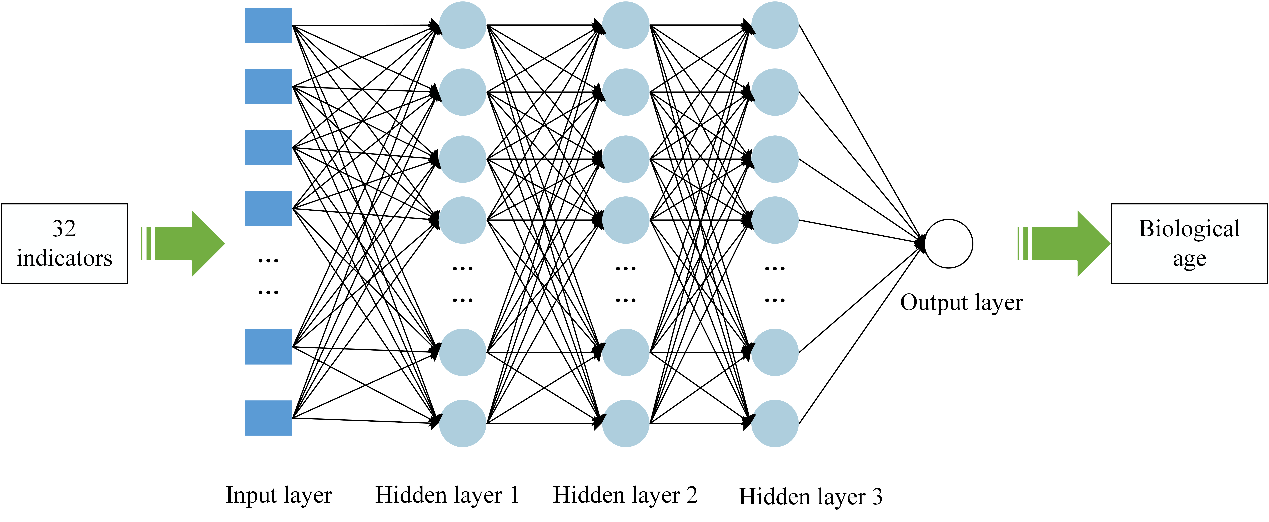


**Figure. DNN Model Diagram**

(6) Model performance was quantified using the Pearson correlation coefficient (*r*) and mean absolute error (*MAE*) between predicted and chronological age in the test set. The larger the r, the smaller the *MAE*, indicating a better model. We calculated $r$ and $MAE$for each model using the follow formula:

$r=\frac{\sum_{i=1}^{n} (x_{i}-\overline{x})(y_{i}-\overline{y})}{\sqrt{\sum_{i=1}^{n} {(x_{i}-\overline{x})}^{2}}\sqrt{\sum_{i=1}^{n} {(y_{i}-\overline{y})}^{2}}}$ ,

where $n$ is the number of test set, $x_{i}$ is real value and $\overline{x}$ is the mean of *x*, $y_{i}$ is the predicted value and $\overline{y}$ is the mean of *y*.

$MAE\left( y,\hat{y} \right)=\frac{1}{n}\sum_{i=0}^{n-1} |y_{i}-\hat{y}_{i}|$ ,

where $n$ is the number of test set, $y_{i}$ is the true value, $\hat{y}_{i}$ is the predict value.

(7) Input the 32 indicator data from three health checkups for each individual into the corresponding trained DNN models, and the predicted BA will be output.

All models were implemented in Python 3.7.5.

3. Biological Age models assessment

In this study, chronological age could be predicted with modest accuracy for BA (females in the first checkup, Pearson’s r = 0.670, MAE = 6.451 years; females in the second checkup, Pearson’s r = 0.675, MAE = 6.549 years; females in the third checkup, Pearson’s r = 0.707, MAE = 6.417; males in the first checkup, Pearson’s r = 0.656, MAE = 7.403 years; males in the second checkup, Pearson’s r = 0.652, MAE = 7.391 years; males in the third checkup, Pearson’s r = 0.697, MAE = 7.426; see **Figure** below).


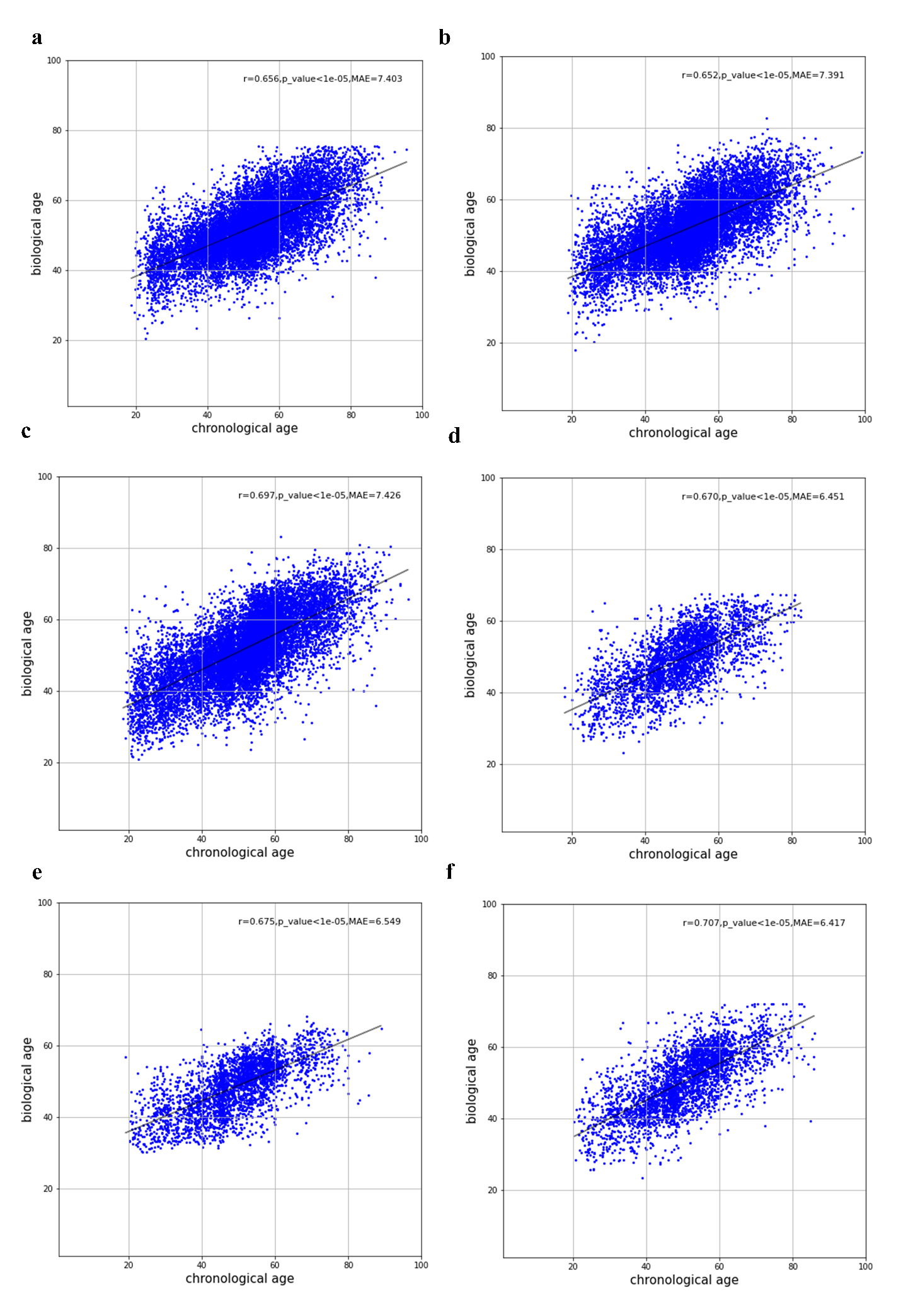


**Figure. Relationship between biological age and chronological age**

Scatterplots show associations between chronological and biological age for prediction models. Lines of best fit are indicated with solid black lines. **a, b, and c** show results for the male test set for the first, second, and third checkup; **d, e, and f** show results for the female test set for the first, second, and third checkup. r = Pearson correlation coefficient. MAE = Mean absolute error.

**References**

1. Putin E, Mamoshina P, Aliper A, et al. Deep biomarkers of human aging: Application of deep neural networks to biomarker development. Aging (Albany NY). 2016;8(5):1021-1033. doi:10.18632/aging.100968

2. Mamoshina P, Kochetov K, Putin E, et al. Population Specific Biomarkers of Human Aging: A Big Data Study Using South Korean, Canadian, and Eastern European Patient Populations. J Gerontol A Biol Sci Med Sci. 2018;73(11):1482-1490. doi:10.1093/gerona/gly005

3. Bernard D, Doumard E, Ader I, Kemoun P, Pagès JC, Galinier A, et al. Explainable machine learning framework to predict personalized physiological aging. Aging Cell. 2023 Aug;22(8):e13872. doi: 10.1111/acel.13872

4. Tian YE, Cropley V, Maier AB, Lautenschlager NT, Breakspear M, Zalesky A. Heterogeneous aging across multiple organ systems and prediction of chronic disease and mortality. Nat Med. 2023;29(5):1221-1231. doi: 10.1038/s41591-023-02296-6

5. Bortz J, Guariglia A, Klaric L, Tang D, Ward P, Geer M, et al. Biological age estimation using circulating blood biomarkers. Commun Biol. 2023;6(1):1089. doi: 10.1038/s42003-023-05456-z.

6. Qiu W, Chen H, Kaeberlein M, Lee SI. ExplaiNAble BioLogical Age (ENABL Age): an artificial intelligence framework for interpretable biological age. Lancet Healthy Longev. 2023;4(12):e711-e723. doi: 10.1016/S2666-7568(23)00189-7.
