# Supplementary material for: Association of clinical biomarker-based biological age and aging trajectory with cardiovascular disease and all-cause mortality in Chinese adults: a population-based cohort study
Source: BMC Public Health. 2025 Mar 4;25:868. doi: 10.1186/s12889-025-22114-7 (PMC11881332; doi:10.1186/s12889-025-22114-7)
Supplement: Supplementary file 3 — Supplementary Material 3. [file 12889_2025_22114_MOESM3_ESM.docx]

**Supplemental Material: Tables S1-S4**

**Table S1. Characteristics of 40 071 participants in the third checkup according to the aging trajectories**

| Characteristic | Low-stable (n=9728) | Increasing-  Decreasing (n=4379) | Low-increasing (n=5957) | High-decreasing (n=5858) | Decreasing-increasing (n=3033) | High-stable (n=11 116) | *P* value |
| --- | --- | --- | --- | --- | --- | --- | --- |
| CA, mean (SD), y^*^ | 52.50 (11.72) | 52.43 (11.73) | 52.61 (11.69) | 52.55 (11.70) | 52.25 (11.90) | 52.66 (11.75) | 0.6038 |
| BA, mean (SD), y^*^ | 46.15 (7.38) | 47.90 (7.10) | 56.10 (7.01) | 47.45 (6.95) | 55.58 (7.21) | 56.59 (7.49) | <0.0001 |
| Sex, n (%) |  |  |  |  |  |  |  |
| Female | 2352 (24.18) | 1053 (24.05) | 1341 (22.51) | 1265 (21.59) | 773 (25.49) | 2683 (24.14) | <0.0001 |
| Male | 7376 (75.82) | 3326 (75.95) | 4616 (77.49) | 4593 (78.41) | 2260 (74.51) | 8433 (75.86) |  |
| Education level, n (%) |  |  |  |  |  |  |  |
| High school or below | 8460 (87.22) | 3957 (90.76) | 5220 (87.83) | 5313 (90.99) | 2773 (91.67) | 10 120 (91.25) | <0.0001 |
| College or above | 1240 (12.78) | 403 (9.24) | 723 (12.17) | 526 (9.01) | 252 (8.33) | 971 (8.75) |  |
| Missing | 28 | 19 | 14 | 19 | 8 | 25 |  |
| Occupation, n (%) |  |  |  |  |  |  |  |
| Coal miner | 3155 (32.50) | 1340 (30.73) | 1681 (28.28) | 2144 (36.72) | 958 (31.63) | 2971 (26.79) | <0.0001 |
| Others | 6554 (67.50) | 3020 (69.27) | 4263 (71.72) | 3695 (63.28) | 2071 (68.37) | 8118 (73.21) |  |
| Missing | 19 | 19 | 13 | 19 | 4 | 27 |  |
| Physical activity, n (%) |  |  |  |  |  |  |  |
| Low-intensity | 3457 (35.59) | 1383 (31.69) | 1919 (32.26) | 2096 (35.87) | 1007 (33.25) | 3479 (31.35) | <0.0001 |
| Moderate-intensity | 4886 (50.30) | 2363 (54.15) | 3247 (54.59) | 2881 (49.31) | 1622 (53.55) | 5987 (53.96) |  |
| High-intensity | 1370 (14.10) | 618 (14.16) | 782 (13.15) | 866 (14.82) | 400 (13.21) | 1630 (14.69) |  |
| Missing | 15 | 15 | 9 | 15 | 4 | 20 |  |
| Smoking status, n (%) |  |  |  |  |  |  |  |
| Never | 6049 (62.28) | 2714 (62.20) | 3683 (61.92) | 3448 (59.01) | 1877 (61.97) | 6794 (61.22) | 0.0006 |
| Quit | 417 (4.29) | 156 (3.58) | 255 (4.29) | 256 (4.38) | 113 (3.73) | 519 (4.68) |  |
| Current | 3247 (33.43) | 1493 (34.22) | 2010 (33.79) | 2139 (36.61) | 1039 (34.30) | 3785 (34.11) |  |
| Missing | 15 | 16 | 9 | 15 | 4 | 18 |  |
| Alcohol consumption, n (%) |  |  |  |  |  |  |  |
| Non-drinker | 6360 (65.49) | 2837 (65.02) | 3890 (65.41) | 3634 (62.19) | 1947 (64.28) | 7114 (64.13) | <0.0001 |
| Quit | 46 (0.47) | 6 (0.14) | 20 (0.34) | 41 (0.70) | 16 (0.53) | 69 (0.62) |  |
| Current drinker | 3305 (34.03) | 1520 (34.84) | 2037 (34.25) | 2168 (37.10) | 1066 (35.19) | 3910 (35.25) |  |
| Missing | 17 | 16 | 10 | 15 | 4 | 23 |  |
| Salt consumption habit, n (%) |  |  |  |  |  |  |  |
| Prefer less salty | 1868 (19.24) | 746 (17.10) | 896 (15.07) | 1070 (18.32) | 510 (16.84) | 1891 (17.04) | <0.0001 |
| Medium | 6778 (69.80) | 3089 (70.80) | 4466 (75.10) | 4139 (70.85) | 2239 (73.92) | 8126 (73.23) |  |
| Prefer salty | 1065 (10.97) | 528 (12.10) | 585 (9.84) | 633 (10.84) | 280 (9.24) | 1080 (9.73) |  |
| Missing | 17 | 16 | 10 | 16 | 4 | 19 | Missing |
| Self-reported hypertension history, n (%) |  |  |  |  |  |  |  |
| No | 9001 (92.76) | 3830 (87.78) | 5210 (87.62) | 5054 (86.53) | 2616 (86.45) | 8778 (79.14) | <0.0001 |
| Yes | 703 (7.24) | 533 (12.22) | 736 (12.38) | 787 (13.47) | 410 (13.55) | 2314 (20.86) |  |
| Missing | 24 | 16 | 11 | 17 | 7 | 24 |  |
| Self-reported diabetes history, n (%) |  |  |  |  |  |  |  |
| No | 9549 (98.31) | 4209 (96.45) | 5724 (96.23) | 5622 (96.25) | 2932 (96.80) | 10 322 (93.02) | <0.0001 |
| Yes | 164 (1.69) | 155 (3.55) | 224 (3.77) | 219 (3.75) | 97 (3.20) | 774 (6.98) |  |
| Missing | 15 | 15 | 9 | 17 | 4 | 20 |  |
| Self-reported dyslipidemia history, n (%) |  |  |  |  |  |  |  |
| No | 9296 (95.71) | 4170 (95.55) | 5685 (95.58) | 5530 (94.68) | 2890 (95.41) | 10 456 (94.22) | <0.0001 |
| Yes | 417 (4.29) | 194 (4.45) | 263 (4.42) | 311 (5.32) | 139 (4.59) | 642 (5.78) |  |
| Missing | 15 | 15 | 9 | 17 | 4 | 18 |  |
| Antihypertensive drug, n (%) |  |  |  |  |  |  |  |
| No | 9199 (95.13) | 3970 (91.75) | 5410 (91.71) | 5228 (90.25) | 2711 (90.31) | 9288 (84.88) | <0.0001 |
| Yes | 471 (4.87) | 357 (8.25) | 489 (8.29) | 565 (9.75) | 291 (9.69) | 1654 (15.12) |  |
| Missing | 58 | 52 | 58 | 65 | 31 | 174 |  |
| Antidiabetic drug, n (%) |  |  |  |  |  |  |  |
| No | 9574 (98.70) | 4224 (97.30) | 5760 (97.23) | 5666 (97.52) | 2952 (97.88) | 10 440 (94.78) | <0.0001 |
| Yes | 126 (1.30) | 117 (2.70) | 164 (2.77) | 144 (2.48) | 64 (2.12) | 575 (5.22) |  |
| Missing | 28 | 38 | 33 | 48 | 17 | 101 |  |
| Lipid-lowering drug, n (%) |  |  |  |  |  |  |  |
| No | 9670 (99.40) | 4346 (99.25) | 5924 (99.45) | 5818 (99.32) | 3013 (99.34) | 10 999 (98.95) | 0.0010 |
| Yes | 58 (0.60) | 33 (0.75) | 33 (0.55) | 40 (0.68) | 20 (0.66) | 117 (1.05) |  |

^*^ The analysis of variance was used to examine the differences of characteristics among aging trajectory groups; others used chi-square test.

Low-stable refers to a persistent low level aging state; Increasing-decreasing denotes an aging pattern that begins with low level aging status, followed by an upward and downward trajectory; Low-increasing indicates an aging trajectory beginning with low aging status and then continually increasing; High-decreasing denotes an aging trajectory beginning with a high degree of aging status, followed by persistent decline; Decreasing-increasing signifies an aging trajectory beginning with a high degree of aging status, followed by decline and then rise again; High-stable implies maintaining a persistently high state of aging trajectory.

Data are n (%), except where otherwise stated.

CA indicates chronological age; BA, biological age; SD, standard deviation.

**Table S2. Trends of 32 indicators during checkup time in the low-increasing and high-decreasing groups, and the differences between them**

| Indicators | Low-increasing group (n=5957) | | |  | High-decreasing group (n=5858) | | | *P* interaction**^†^** |
| --- | --- | --- | --- | --- | --- | --- | --- | --- |
|  | Model type^*^ | *β (95% CI)* | *P* value |  | Model type^*^ | *β (95% CI)* | *P* value |  |
| SBP, mmHg | Random Coefficients Model | 5.37(5.15 ~ 5.59) | <0.0001 |  | Random Intercept Model | -3.04(-3.26 ~ -2.82) | <0.0001 | <0.0001 |
| DBP, mmHg | Random Intercept Model | 1.48(1.32 ~ 1.64) | <0.0001 |  | Random Intercept Model | 0.22(0.08 ~ 0.36) | 0.0032 | <0.0001 |
| BMI, kg/m^2^ | Random Intercept Model | -0.04(-0.08 ~ 0.00) | 0.0174 |  | Random Coefficients Model | 0.03(-0.01 ~ 0.07) | 0.0273 | 0.0010 |
| WHR | Random Intercept Model | 0.01(0.01 ~ 0.01) | <0.0001 |  | Random Intercept Model | -0.00(0.00 ~ 0.00) | 0.0344 | <0.0001 |
| FBG, mmol/L | Random Coefficients Model | 0.20(0.18 ~ 0.22) | <0.0001 |  | Random Intercept Model | 0.08(0.06 ~ 0.10) | <0.0001 | <0.0001 |
| TC, mmol/L | Random Intercept Model | 0.09(0.07 ~ 0.11) | <0.0001 |  | Random Intercept Model | 0.00(-0.01 ~ 0.02) | 0.8247 | <0.0001 |
| TG, mmol/L | Random Intercept Model | -0.08(-0.1 ~ -0.06) | <0.0001 |  | Random Coefficients Model | 0.07(0.05 ~ 0.09) | <0.0001 | <0.0001 |
| HDL-C, mmol/L | Random Coefficients Model | 0.01(0.00 ~ 0.02) | <0.0001 |  | Random Coefficients Model | -0.04(-0.05 ~ -0.04) | <0.0001 | <0.0001 |
| LDL-C, mmol/L | Random Intercept Model | 0.19(0.17 ~ 0.21) | <0.0001 |  | Random Intercept Model | 0.11(0.09 ~ 0.12) | <0.0001 | <0.0001 |
| ALT, U/L | Random Intercept Model | -1.98(-2.22 ~ -1.74) | <0.0001 |  | Random Coefficients Model | 0.83(0.58 ~ 1.08) | <0.0001 | <0.0001 |
| TBIL, μmol/L | Random Intercept Model | 0.50(0.42 ~ 0.58) | <0.0001 |  | Random Intercept Model | 0.28(0.20 ~ 0.36) | <0.0001 | 0.0002 |
| Cr, μmol/L | Random Intercept Model | -4.37(-4.68 ~ -4.06) | <0.0001 |  | Random Intercept Model | -5.21(-5.54 ~ -4.88) | <0.0001 | 0.0003 |
| BUN, mmol/L | Random Coefficients Model | 0.22(0.20 ~ 0.24) | <0.0001 |  | Random Intercept Model | -0.08(-0.10 ~ -0.06) | <0.0001 | <0.0001 |
| UA, μmol/L | Random Coefficients Model | 1.81(0.83 ~ 2.79) | 0.0003 |  | Random Coefficients Model | 2.16(1.06 ~ 3.26) | 0.0001 | 0.6499 |
| hs-CRP, mg/L | Random Intercept Model | 0.30(0.22 ~ 0.38) | <0.0001 |  | Random Intercept Model | -0.02(-0.09 ~ 0.06) | 0.7145 | <0.0001 |
| WBC, ×10^9^ /L | Random Intercept Model | -0.09(-0.11 ~ -0.07) | <0.0001 |  | Random Intercept Model | -0.08(-0.10 ~ -0.06) | <0.0001 | 0.3170 |
| NEUT, ×10^9^ /L | Random Intercept Model | 0.05(0.03 ~ 0.07) | <0.0001 |  | Random Coefficients Model | -0.01(-0.03 ~ 0.01) | 0.2381 | <0.0001 |
| LYM, ×10^9^ /L | Random Intercept Model | -0.13(-0.14 ~ -0.12) | <0.0001 |  | Random Intercept Model | -0.06(-0.07 ~ -0.06) | <0.0001 | <0.0001 |
| MON, ×10^9^ /L | Random Intercept Model | -0.03(-0.03 ~ -0.03) | <0.0001 |  | Random Intercept Model | -0.01(-0.01 ~ -0.01) | <0.0001 | <0.0001 |
| NEUT% | Random Intercept Model | 1.76(1.64 ~ 1.88) | <0.0001 |  | Random Intercept Model | 0.56(0.44 ~ 0.68) | <0.0001 | <0.0001 |
| LYM% | Random Intercept Model | -1.64(-1.74 ~ -1.54) | <0.0001 |  | Random Intercept Model | -0.59(-0.69 ~ -0.49) | <0.0001 | <0.0001 |
| MON% | Random Intercept Model | -0.40(-0.44 ~ -0.36) | <0.0001 |  | Random Intercept Model | -0.16(-0.20 ~ -0.12) | <0.0001 | <0.0001 |
| RBC, ×10^12^/L | Random Intercept Model | -0.08(-0.08 ~ -0.07) | <0.0001 |  | Random Intercept Model | -0.01(-0.01 ~ 0.00) | 0.0112 | <0.0001 |
| HCT | Random Intercept Model | 0.00(0.00 ~ 0.00) | <0.0001 |  | Random Intercept Model | 0.00(0.00 ~ 0.01) | <0.0001 | <0.0001 |
| MCV, fL | Random Intercept Model | 1.04(0.96 ~ 1.12) | <0.0001 |  | Random Intercept Model | 0.48(0.40 ~ 0.56) | <0.0001 | <0.0001 |
| HGB, g/L | Random Intercept Model | -0.92(-1.10 ~ -0.74) | <0.0001 |  | Random Intercept Model | 0.13(-0.05 ~ 0.31) | 0.1764 | <0.0001 |
| MCH, pg | Random Intercept Model | 0.30(0.28 ~ 0.32) | <0.0001 |  | Random Intercept Model | 0.08(0.04 ~ 0.12) | <0.0001 | <0.0001 |
| MCHC, g/L | Random Intercept Model | -0.80(-1.15 ~ -0.45) | <0.0001 |  | Random Intercept Model | -1.14(-1.49 ~ -0.79) | <0.0001 | 0.2075 |
| PLT, ×10^9^ /L | Random Coefficients Model | 2.20(1.51 ~ 2.89) | <0.0001 |  | Random Coefficients Model | 9.63(8.94 ~ 10.32) | <0.0001 | <0.0001 |
| MPV, fL | Random Coefficients Model | 0.31(0.29 ~ 0.33) | <0.0001 |  | Random Coefficients Model | 0.16(0.14 ~ 0.18) | <0.0001 | <0.0001 |
| PCT | Random Intercept Model | 0.01(0.01 ~ 0.01) | <0.0001 |  | Random Intercept Model | 0.01(0.01 ~ 0.01) | <0.0001 | 0.0814 |
| PDW, % | Random Coefficients Model | -0.43(-0.45 ~ -0.41) | <0.0001 |  | Random Coefficients Model | -0.22(-0.26 ~ -0.18) | <0.0001 | <0.0001 |

^*^ Model type is determined by Wald Z-tests of covariance parameters to decide whether a Random Intercept Model or a Random Coefficients Model is more appropriate. Selecting a Random Intercept Model means that, although there are differences in the intercepts of these indicators among individuals over checkup time, the slopes are regarded as the same. This suggests that the way these indicators change over time across individuals shows no significant difference. In contrast, choosing a Random Coefficients Model implies that both the intercepts and the slopes vary among individuals, suggesting a statistically significant difference in the trends of these indicators over time across individuals.

**^†^** *P* interaction <0.05 indicates that there is a statistically significant difference in the changing trends of the indicators among groups.

ALT indicates Alanine aminotransferase; BMI indicates Body mass index; BUN indicates Blood urea nitrogen; Cr indicates Creatinine; DBP indicates Diastolic blood pressure; FBG indicates Fasting blood glucose; HCT indicates Hematocrit; HDL-C indicates High density lipoprotein cholesterol; HGB indicates Hemoglobin; hs-CRP indicates Hypersensitive C-reactive protein; IQR indicates Interquartile range; LDL-C indicates Low density lipoprotein cholesterol; LYM indicates Lymphocyte; LYM% indicates the percentage of lymphocyte; MCH indicates Mean corpuscular hemoglobin; MCHC indicates Mean corpuscular hemoglobin concentration; MCV indicates Mean corpuscular volume; MON indicates Monocytes; MON% indicates the percentage of monocytes; MPV indicates Mean platelet volume; NEUT indicates Neutrophil count; NEUT% indicates the percentage of neutrophil; PCT indicates Platelet crit; PDW indicates Platelet distribution width; PLT indicates Platelet count; RBC indicates red blood cell count; SBP indicates Systolic blood pressure; SD indicates Standard deviation; TBIL indicates Total bilirubin; TC indicates Total cholesterol; TG indicates Triglyceride; UA indicates Uric acid; WBC indicates White blood cell count; WHR indicates Waist-to-hip ratio.

**Table S3. Sensitivity analyses: Association of baseline aging status with the risk of cardiovascular disease and all-cause mortality**

| **Outcome** | **Baseline aging status** | **Cases/incident rate^*^** | **Adjusted HR (95% CI)** |
| --- | --- | --- | --- |
| **Sensitivity analysis 1: Excluding participants with missing values for observed covariates^†^** | | | |
| Cardiovascular disease  (n=69 028) | Decelerated aging | 1282 (5.22) | 0.84 (0.79-0.90) |
|  | Normal aging | 3015 (6.35) | Ref |
|  | Accelerated aging | 1712 (7.49) | 1.16 (1.09-1.23) |
| All-cause mortality  (n=69 028) | Decelerated aging | 1959 (7.75) | 0.86 (0.81-0.91) |
|  | Normal aging | 4445 (9.06) | Ref |
|  | Accelerated aging | 2538 (10.68) | 1.17 (1.12-1.23) |
| **Sensitivity analysis 2: Excluding participants with incident CVD or death within the initial two years of follow-up^†^** | | | |
| Cardiovascular disease  (n=74 447) | Decelerated aging | 1273 (4.87) | 0.84 (0.79-0.89) |
|  | Normal aging | 3116 (6.02) | Ref |
|  | Accelerated aging | 1821 (7.18) | 1.17（1.11-1.24） |
| All-cause mortality  (n=75 128) | Decelerated aging | 2079 (7.72) | 0.86 (0.82-0.90) |
|  | Normal aging | 4935 (9.2) | Ref |
|  | Accelerated aging | 2894 (10.92) | 1.18 (1.12-1.23) |
| **Sensitivity analysis 3: Excluding participants with a self-reported disease history and medication usage^#^** | | | |
| Cardiovascular disease  (n=64 484) | Decelerated aging | 1169 (4.92) | 0.82 (0.77-0.88) |
|  | Normal aging | 2632 (5.91) | Ref |
|  | Accelerated aging | 1432 (6.83) | 1.16 (1.09-1.23) |
| All-cause mortality (n=64 484) | Decelerated aging | 1789 (7.25) | 0.86 (0.81-0.91) |
|  | Normal aging | 3860 (8.17) | Ref |
|  | Accelerated aging | 2159 (9.63) | 1.17 (1.11-1.24) |
| **Sensitivity analysis 4: Association of baseline aging status with the risk of cardiovascular disease treating non-CVD deaths as competing risk events^†^** | | | |
|  | Decelerated aging |  | 0.85 (0.80-0.91) |
|  | Normal aging |  | Ref |
|  | Accelerated aging |  | 1.15 (1.09-1.22) |

^*^Incident rate indicates per 1000 person-years.

**^†^**Models were adjusted for chronological age, sex, education level, occupation, physical activity, smoking status, alcohol consumption, salt consumption habit, self-reported hypertension history, self-reported diabetes history, self-reported dyslipidemia history, antihypertensive drug, antidiabetic drug, and lipid-lowering drug.

^#^Models were adjusted for chronological age, sex, education level, occupation, physical activity, smoking status, alcohol consumption, salt consumption habit.

CI indicates confidence interval; HR, hazard ratio; Ref, reference.

**Table S4. Sensitivity analyses: Association of aging trajectories with the risk of cardiovascular disease and all-cause mortality**

| **Outcome** | **Aging trajectory groups^*^** | **Cases/incident rate^†^** | **Adjusted HR (95% CI)** |
| --- | --- | --- | --- |
| **Sensitivity analysis 1: Excluding participants with missing values for observed covariates^#^** | | | |
| Cardiovascular disease  (n=39 386) | Low-stable | 491 (4.83) | Ref |
|  | Increasing-decreasing | 256 (5.70) | 1.14 (0.98-1.32) |
|  | Low-increasing | 390 (6.44) | 1.31 (1.14-1.49) |
|  | High-decreasing | 368 (6.12) | 1.21 (1.05-1.38) |
|  | Decreasing-increasing | 208 (6.72) | 1.39 (1.18-1.63) |
|  | High-stable | 895 (8.09) | 1.55 (1.38-1.73) |
| All-cause mortality  (n=39 386) | Low-stable | 608 (5.87) | Ref |
|  | Increasing-decreasing | 330 (7.18) | 1.23 (1.07-1.40) |
|  | Low-increasing | 446 (7.17) | 1.20 (1.06-1.35) |
|  | High-decreasing | 438 (7.11) | 1.23 (1.08-1.39) |
|  | Decreasing-increasing | 242 (7.61) | 1.35 (1.16-1.56) |
|  | High-stable | 1090 (9.53) | 1.56 (1.41-1.73) |
| **Sensitivity analysis 2: Excluding participants with incident CVD or death within the initial two years of follow-up^#^** | | | |
| Cardiovascular disease  (n=39 409) | Low-stable | 447 (4.37) | Ref |
|  | Increasing-decreasing | 233 (5.10) | 1.15 (0.98-1.35) |
|  | Low-increasing | 340 (5.54) | 1.27 (1.10-1.47) |
|  | High-decreasing | 331 (5.42) | 1.20 (1.04-1.38) |
|  | Decreasing-increasing | 185 (5.91) | 1.37 (1.16-1.63) |
|  | High-stable | 818 (7.23) | 1.61 (1.43-1.80) |
| All-cause mortality  (n=39 724) | Low-stable | 559 (5.35) | Ref |
|  | Increasing-decreasing | 301 (6.43) | 1.20 (1.05-1.39) |
|  | Low-increasing | 391 (6.19) | 1.14 (1.00-1.30) |
|  | High-decreasing | 402 (6.42) | 1.21 (1.06-1.37) |
|  | Decreasing-increasing | 212 (6.57) | 1.27 (1.09-1.49) |
|  | High-stable | 1022 (8.73) | 1.59 (1.42-1.75) |
| **Sensitivity analysis 3: Excluding participants with a self-reported disease history and medication usage^$^** | | | |
| Cardiovascular disease  (n=32 806) | Low-stable | 396 (4.30) | Ref |
|  | Increasing-decreasing | 201 (5.20) | 1.23 (1.04-1.46) |
|  | Low-increasing | 296 (5.73) | 1.35 (1.16-1.57) |
|  | High-decreasing | 270 (5.32) | 1.22 (1.05-1.43) |
|  | Decreasing-increasing | 152 (5.84) | 1.40 (1.16-1.69) |
|  | High-stable | 590 (7.01) | 1.64 (1.44-1.86） |
| All-cause mortality (n=32 806) | Low-stable | 496 (5.30) | Ref |
|  | Increasing-decreasing | 257 (6.51) | 1.27 (1.09-1.47) |
|  | Low-increasing | 348 (6.59) | 1.23 (1.07-1.41) |
|  | High-decreasing | 323 (6.23) | 1.20 (1.04-1.38) |
|  | Decreasing-increasing | 195 (7.30) | 1.42 (1.20-1.67) |
|  | High-stable | 761 (8.79) | 1.64 (1.46-1.83) |
| **Sensitivity analysis 4: Association of aging trajectories with the risk of cardiovascular disease treating non-CVD deaths as competing risk events^#^** | | | |
|  | Low-stable |  | Ref |
|  | Increasing-decreasing |  | 1.14 (0.98-1.33) |
|  | Low-increasing |  | 1.30 (1.14-1.48) |
|  | High-decreasing |  | 1.21 (1.06-1.38) |
|  | Decreasing-increasing |  | 1.37 (1.16-1.61) |
|  | High-stable |  | 1.57 (1.41-1.75) |
| **Sensitivity analysis 5: Excluding participants with missing biological age at any checkup^#^** | | | |
| Cardiovascular disease  (n=27 967) | Low-stable | 346 (4.86) | Ref |
|  | Increasing-decreasing | 202 (5.58) | 1.16 (0.98-1.38) |
|  | Low-increasing | 255 (6.56) | 1.34 (1.14-1.58) |
|  | High-decreasing | 235 (5.93) | 1.18 (1.00-1.40) |
|  | Decreasing-increasing | 186 (6.62) | 1.38 (1.15-1.65) |
|  | High-stable | 638 (8.27) | 1.64 (1.44-1.87) |
| All-cause mortality  (n=27 967) | Low-stable | 411 (5.67) | Ref |
|  | Increasing-decreasing | 249 (6.72) | 1.22 (1.04-1.43) |
|  | Low-increasing | 289 (7.22) | 1.26 (1.08-1.46) |
|  | High-decreasing | 275 (6.77) | 1.21 (1.04-1.41) |
|  | Decreasing-increasing | 205 (7.09) | 1.28 (1.08-1.51) |
|  | High-stable | 731 (9.16) | 1.57 (1.39-1.78) |

^*^Incident rate indicates per 1000 person-years.

**^†^**Low-stable refers to a persistent low level aging state; Increasing-decreasing denotes an aging pattern that begins with low level aging status, followed by an upward and downward trajectory; Low-increasing indicates an aging trajectory beginning with low aging status and then continually increasing; High-decreasing denotes an aging trajectory beginning with a high degree of aging status, followed by persistent decline; Decreasing-increasing signifies an aging trajectory beginning with a high degree of aging status, followed by decline and then rise again; High-stable implies maintaining a persistently high state of aging trajectory.

^#^Models were adjusted for chronological age, sex, education level, occupation, physical activity, smoking status, alcohol consumption, salt consumption habit, self-reported hypertension history, self-reported diabetes history, self-reported dyslipidemia history, antihypertensive drug, antidiabetic drug, and lipid-lowering drug.

^$^Models were adjusted for chronological age, sex, education level, occupation, physical activity, smoking status, alcohol consumption, salt consumption habit.

CI indicates confidence interval; HR, hazard ratio; Ref, reference.
